# An Eye-Tracking Investigation of Written Sarcasm Comprehension: The Roles of Familiarity and Context

**DOI:** 10.1037/xlm0000285

**Published:** 2016-08-08

**Authors:** Alexandra Țurcan, Ruth Filik

**Affiliations:** 1 School of Psychology, University of Nottingham

**Keywords:** sarcasm, irony, language comprehension, figurative language, eye-tracking

## Abstract

This article addresses a current theoretical debate between the standard pragmatic model, the graded salience hypothesis, and the implicit display theory, by investigating the roles of the context and of the properties of the sarcastic utterance itself in the comprehension of a sarcastic remark. Two eye-tracking experiments were conducted where we manipulated the speaker’s expectation in the context and the familiarity of the sarcastic remark. The results of the first eye-tracking study showed that literal comments were read faster than unfamiliar sarcastic comments, regardless of whether an explicit expectation was present in the context. The results of the second eye-tracking study indicated an early processing difficulty for unfamiliar sarcastic comments, but not for familiar sarcastic comments. Later reading time measures indicated a general difficulty for sarcastic comments. Overall, results seem to suggest that the familiarity of the utterance does indeed affect the time course of sarcasm processing (supporting the graded salience hypothesis), although there is no evidence that making the speaker’s expectation explicit in the context affects it as well (thus failing to support the implicit display theory).

Verbal irony and sarcasm are forms of nonliteral language that are commonly used in our everyday interactions. Gibbs (2000) and Hancock (2004) both reported similar rates of ironic language use—about 8% of conversational turns include an ironic comment, be it between friends, or total strangers. However, psycholinguists have found it difficult to define these two forms of figurative language and conceptualise the mechanisms through which people manage to understand and make use of them in their everyday life (Bryant, 2012).

## Operational Definitions

*Irony* is defined as a form of indirect language, used when the speaker expresses one evaluative utterance but implies a different evaluative appraisal (Burgers, van Mulken, & Schellens, 2011). An example of an ironic comment is No. 1 below, where the expressed evaluation is that the weather is “great” but the implied evaluation is that the weather is “terrible.” Sarcasm is a specific form of irony, which is used when the target object of the comment is a person (Kreuz & Glucksberg, 1989). An example of a sarcastic comment is No. 2 below, where the expressed evaluation is that the colleague is “early” but the implied evaluation is that they are “late.”

1 *Nonsarcastic irony*—Uttering while standing outside in the pouring rain: “The weather is great today!”
2 *Sarcastic irony*—Uttering to a colleague who arrived at a meeting half an hour late: “You’re early!”

This article is concerned with the comprehension of sarcastic irony (one of the most commonly used forms of irony), where the expressed (positive) evaluation is the direct opposite of the intended (negative) evaluation, as in example No. 2 above.

## Current Theoretical Debates

Existing theories of sarcasm processing can be classified into modular accounts and interactive accounts, and they differ in terms of their predictions for the time course of sarcasm processing, and the roles played by the properties of the utterance itself and by contextual factors. In what follows, we give a brief overview of the two theoretical categories, and the reported experiments focus on testing the predictions of specific theories from each category.

*Modular accounts* claim that the literal meaning of a sarcastic utterance is usually accessed first and the sarcastic meaning is accessed afterward. One example of a well-known modular account is the *standard pragmatic model* (Grice, 1975), which predicts that sarcastic utterances will always take longer to process than the same utterances used literally, because they will always require the extra step of processing and then rejecting the literal meaning of the sarcastic utterance, irrespective of how supportive the context is (Gibbs, 1999). A more recent modular account is *the graded salience hypothesis* (Giora, 1997, 2003), which introduces the concept of salience. A salient meaning is one that is stored in the mental lexicon due to its familiarity, conventionality, frequency, or prototypicality (Peleg, Giora, & Fein, 2001). According to the graded salience hypothesis, salient meanings are processed first, regardless of strength of context (Giora, 1997).

A familiar sarcastic remark (like, *“That’s great!”*) is assumed to have two salient meanings: the sarcastic and the nonsarcastic, and they will both be activated in parallel. Therefore, the graded salience hypothesis predicts that familiar sarcastic remarks should not have longer processing times than their literal counterparts. An unfamiliar sarcastic remark however, has only one salient meaning, which is usually the literal one. In the case of unfamiliar sarcastic remarks, the graded salience hypothesis predicts a very similar comprehension process to the standard pragmatic model—unfamiliar sarcastic comments will take longer to process compared with literal counterparts, because the salient literal meaning will be activated first, followed by the nonsalient sarcastic meaning (Giora, 1997, 2003). Diverging from the standard pragmatic model, however, the salience-based interpretation will not be discarded, because it contributes to the interpretation process.

*Interactive accounts* claim that the sarcastic meaning is accessed directly in supportive contexts. A classic example is *the direct access view* (Gibbs, 1986), which predicts that sarcastic utterances should be processed in equal time to their literal counterparts when embedded in supportive contexts (that is, contexts where there is a discrepancy between expectation and reality), because readers do not have to perform a complete analysis of the literal meaning first (Gibbs & Colston, 2012). A more recent interactive account is the *parallel constraint satisfaction model* (Pexman, 2008). This is a more general model, that allows for many different and unspecified contextual factors to act as cues for sarcasm and therefore facilitate sarcasm processing. In this respect, the parallel constraint satisfaction model could be considered to be a framework theory, in that it does not have a specific set of factors for which it makes testable predictions.

A testable interactive theory of sarcasm processing specifically (and not figurative language in general), is *the implicit display theory* (Utsumi, 2000) which expands on the direct access view’s claim that context can aid sarcasm comprehension, but dissociates itself from the idea that only one factor can influence sarcasm comprehension. The implicit display theory postulates that sarcasm requires an ironic environment, which is a property of the context. An ironic environment includes three components: (a) the speaker has to have an expectation (known to both interlocutors), (b) the expectation has to be unmet by the current situation, and (c) the speaker has to have a negative emotional attitude toward the incongruity between expectation and reality (Utsumi, 2000). According to the implicit display theory, sarcastic remarks implicitly display this ironic environment, and they can do so to different degrees.

The implicit display is a property of the ironic utterance; to achieve implicit display, this utterance should (a) allude to the speaker’s expectation, (b) violate at least one of Grice’s pragmatic principles, and (c) indirectly express the speaker’s negative attitude (Utsumi, 2000). According to the implicit display theory, sarcasm comprehension is governed by the concept of prototypicality. A prototypical sarcastic utterance is one that satisfies all three conditions for implicit display. The claim is that prototypical sarcastic utterances that fully satisfy the three requirements of implicit display will have the highest degree of ironicalness (that is, they will be perceived as most ironic). Sarcasm that fails to satisfy one or more of the requirements will have a lower score of ironicalness (that is, they will be perceived as less ironic).

Utsumi (2000) gives a mathematical formula (see Equation 1 below) for degree of ironicalness that contains degree of manifestness as a variable (defined as the explicitness of the speaker’s expectation in the context).

$$\text{d}\left(\text{U}\right)={\text{d}}_{\text{m}}*{\text{d}}_{\text{a}}+\left(\text{1}-{\text{d}}_{\text{m}}\right)*{\text{d}}_{\text{d}}+{\text{d}}_{\text{i}}+{\text{d}}_{\text{e}}$$ 1

This is Equation 1: The mathematical formula for degree of ironicalness according to the Implicit Display Theory *(*Utsumi, 2000). The abbreviations are as follows: d(U) = degree of irony; d_m_ = degree of manifestness; d_a_ = degree of allusion; d_d_ = degree of polarity; d_i_ = degree of pragmatic insincerity; d_e_ = degree of indirect expression of negative attitude.

In the series of experiments presented in this article, d_m_ (the degree of manifestness) was the only factor from the formula that was manipulated. All other factors have been kept constant and at their maximal values (d_a_: all sarcastic comments said the opposite of what the speaker meant, d_d_: polarity of the comments was always positive, that is, only sarcastic criticisms were employed, d_i_: the maxim of quality was the only maxim violated, and d_e_: the same sarcastic cues were used across comments, that is, an exclamation mark at the end), so that the ironic environment and implicit display were prototypical and could only vary with degree of manifestness.

One prediction of the implicit display theory is that more prototypical sarcastic utterances, that is, those that are made in contexts in which the speaker’s expectation is made explicit, will be processed faster than or as fast as their literal counterparts (see Utsumi, 2000). Less prototypical sarcastic remarks, that is, those that are uttered in contexts in which the speaker’s expectation is implicit (and hence harder to infer) will be processed more slowly than literal equivalents.

## Empirical Evidence

The first question that researchers have typically addressed is, Do sarcastic utterances take longer to process than literal ones? In a typical experiment, participants would be presented with scenarios that would end in an utterance that could be interpreted as either literal or sarcastic. On the one hand, evidence from self-paced reading studies (e.g., Giora, 1995; Giora, Fein, & Schwartz, 1998; Spotorno & Noveck, 2014), and eye-tracking studies (e.g., Filik & Moxey, 2010; Kaakinen, Olkoniemi, Kinnari, & Hyönä, 2014) showing that sarcasm comprehension takes longer than literal language comprehension, has been taken to support modular accounts. Other evidence showing that sarcasm can be comprehended as fast as literal language, again from self-paced reading (e.g., Gibbs, 1986), and additionally from visual-world paradigm studies (e.g., Kowatch, Whalen, & Pexman, 2013), has been taken as support for more interactive accounts.

To refine the debate, researchers started to address the question of whether properties of the utterance (e.g., its salience) affect the time course of sarcasm comprehension. Although “salience” is a concept loosely defined within the graded salience hypothesis, researchers have generally equated it with “familiarity,” that is, if a sarcastic utterance is deemed “salient,” that means that that utterance is familiar to readers in its sarcastic interpretation. Overall, research seems to support the graded salience hypothesis prediction that familiar sarcastic utterances are processed in equal time to literal ones, but unfamiliar sarcastic utterances take longer to process than their literal counterparts (e.g., lexical decision task, Giora & Fein, 1999, eye-tracking, Filik, Leuthold, Wallington, & Page, 2014, Experiment 1).

In the literature presented above, contextual factors have not been manipulated at all, other than ensuring that sarcastic utterances were embedded in contexts where there was a mismatch between expectation and reality, whereas literal utterances were embedded in contexts where there was no such mismatch. However, it seems intuitive to assume that contextual factors must play a role in sarcasm comprehension along with properties of the utterance itself, because an utterance can only be interpreted as sarcastic in context (Calmus & Caillies, 2014; however, see Giora et al., 2013, for a different view). Thus, researchers wanted to address a third question of, specifically, whether contextual factors affect the time course of sarcasm comprehension. Evidence for the role of context is also mixed. There are studies that showed that the degree of negativity of the event described in the context does indeed affect the time course of sarcasm comprehension (e.g., reading task, Ivanko & Pexman, 2003), and that this negativity can also interact with the explicitness of the expectation in the context to influence how sarcastic a comment is perceived to be (e.g., Utsumi, 2005). Furthermore, the time course of sarcasm comprehension can also be affected by factors like the occupation of the speaker in the context (Pexman, Ferretti, & Katz, 2000), context incongruity, and relationship between characters (Pexman, Whalen, & Green, 2010). These studies support interactive accounts of sarcasm comprehension by showing that context can indeed affect the time course of sarcasm processing (see also Campbell & Katz, 2012).

On the other hand, there are studies showing that the time course of sarcasm comprehension is not affected by having an expectation made explicit in the context (e.g., Giora, Fein, Kaufman, Eisenberg, & Erez, 2009), or by having a character that is known to be sarcastic in the context (e.g., Giora et al., 2007). These studies support the modular accounts.

In conclusion, evidence in the literature with regards to the time course of sarcasm processing is mixed and conflicting (as also shown by Gibbs & Colston, 2012). Possible reasons for this are that some of the studies described above either lacked a good control over confounding factors (e.g., saliency), they did not have the design required in order to distinguish between the two groups of theories, or the methodology they employed was not sensitive enough to reveal reading time differences between sarcastic and literal comments, or effects of the context.

This article focuses on testing the predictions of three theories: two modular (the standard pragmatic model and the graded salience hypothesis), and one interactive (the implicit display theory, because it makes testable predictions about the way in which specific contextual factors should affect sarcasm comprehension). Therefore, from here onward, the focus of the article shifts to these three specific theories.

The present studies set out to contribute to the debate outlined above, specifically, investigating the roles of familiarity of the comment itself and explicitness of expectation in the context using tightly controlled literal and sarcastic stimuli and a sensitive methodology. The eye-tracking method has been previously used to investigate the effect of familiarity (e.g., Filik et al., 2014), but not of contextual factors. This method is a more sensitive and precise measure of comprehension than simple self-paced reading times, and it also allows us to investigate both early and late effects of our manipulations on sarcasm processing. The target comments in our experiments will be disambiguated by a single word, which will also allow us to distinguish early and late stages of processing which might have been confounded in studies that had disambiguating regions made up of several words.

## Experiment 1

The aim of the first experiment was to investigate the role of the speaker’s expectation on sarcasm processing. Therefore, the explicitness of the speaker’s expectation in the context and the literality of the target utterance were manipulated, while all target utterances were unfamiliar (because the predictions made by the two modular accounts and those made by the implicit display theory are most clearly distinct in cases where the sarcastic utterances are unfamiliar). Specifically, both the standard pragmatic model and the graded salience hypothesis would predict a processing difficulty for unfamiliar sarcastic utterances in both explicit and implicit contexts. In other words, in order to support the two modular theories, we would expect to find a main effect of literality, but no interaction between literality and explicitness. In contrast, the implicit display theory would predict that when the speaker’s expectation is made explicit in the context, sarcastic utterances would be read as fast as literal ones, however, when the expectation is implicit, sarcastic utterances would take longer to read than literal ones. In other words, in order to support the implicit display theory, we would expect to find an interaction between literality and explicitness, with longer reading times for sarcastic than literal comments only in the implicit condition.

### Method

#### Participants

Thirty-two students from the University of Nottingham (*M*_age_ = 18 years and 4 months, *SD* = 6 months, 31 females and 1 male) participated in the experiment. All participants were native English speakers, not diagnosed with any reading disorders, and had normal or corrected-to-normal vision. They received course credit in return for their participation.

#### Materials and design

Twenty-four experimental materials were constructed (see Table 1 for an example and Appendix A for a selection; the full set of items is available from the first author). Each scenario was made up of five sentences, describing an interaction between two characters, and ending with a comment that one character made toward the other one. The first sentence of the context simply introduced the two characters and the situation they were in (e.g., *Dean and Chloe were on holiday in Valencia for a week.*).[Table-anchor tbl1]

The second sentence had two versions, which differed between the explicit and implicit conditions. In the explicit condition, the second sentence contained an explicit expectation of the speaker regarding how the other character should behave, which was known to both characters, as required by the implicit display theory (e.g., *The end of the trip was approaching so Dean asked Chloe to think of something thrilling to do on their last day.*). In the implicit condition, the second sentence of the context did not contain any mention of an expectation (e.g., *Their trip was quickly coming to an end, and they weren’t sure what to do on their final day.*). Results from a stimulus norming test revealed that as intended, participants thought that the materials in the explicit condition created an expectation for how the other character should behave significantly more than the materials in the implicit condition (see Appendix B).

The third sentence contained the outcome of the second character’s behavior and it had two versions, which differed between the literal and sarcastic conditions. In the literal conditions, the outcome fulfilled the expectation mentioned in the previous sentence (e.g., *Chloe suggested they go and watch the Formula 1 race, which was Dean’s favorite sport.*). In the sarcastic conditions, the outcome frustrated the expectation mentioned in the previous sentence (e.g., *Chloe suggested they stay in the hotel and watch TV, which was quite boring.*). Results from a stimulus norming test verified that indeed the materials in the sarcastic condition were perceived as significantly more sarcastic than those in the literal condition (see Appendix B).

The final comment was contained in the fourth sentence (e.g., *“Your suggestion is stirring!” Dean said to her.*). In the literal conditions, the speaker meant what they literally said through the final comment, which had a positive meaning, whereas in the sarcastic conditions, the speaker said the opposite of what they meant, that is, they said something positive in order to convey a negative meaning. All final sarcastic comments were nonconventional, meaning they were not familiar to the readers (as shown by a familiarity stimulus norming test; see Appendix B). The fifth sentence was a wrap-up sentence that concluded the scenario (e.g., *They went out.*). Thus the experiment consisted of a 2 literality (literal vs. sarcastic) × 2 speaker’s expectation (explicit vs. implicit) design, with both factors being within-subject and within-item.

Besides the literality, explicitness, and familiarity stimulus norming tests, two more norming tests were conducted. One verified that the materials were suitable for testing the implicit display theory, that is, they fulfilled the offline predictions of the theory: the reader’s expectation for sarcasm was increased in the explicit condition compared with the implicit condition (see Appendix B). The other test investigated whether the conditions differed in terms of how natural they sound to the reader, and the results indicated the literal materials sounded more natural than the sarcastic ones (see Appendix B). This is perhaps to be expected, given that sarcastic comments are employed significantly less in every day speech than literal ones.

Thirty-six filler materials accompanied the 24 experimental materials. A third of the filler items also contained two characters but ended in a literal negative utterance, another third did not have any characters and were informative texts, whereas the final third contained two characters and ended in a literal positive utterance (see a selection of filler items in Appendix C).

The software used to display the texts (Eye Track; http://blogs.umass.edu/eyelab/software/) ensured the randomization and counterbalancing of the scenarios. For each scenario, there were four stimulus presentation files, each containing only one version of each scenario, and a total of six experimental items for each condition. Each participant was presented with one stimulus file, so that in the end data were collected from eight participants for each stimulus file. The order in which the scenarios were presented within each stimulus file was randomized for each participant.

#### Procedure

Eye movements were recorded via an SR Research Eyelink 1000 eye tracker that sampled eye position every millisecond. Viewing was binocular, but only one eye was recorded for each participant. Materials were displayed on a computer screen approximately 56 cm from participants’ eyes. Before the start of the experiment, the procedure was explained to the participants. They were instructed to read as they would normally, taking as much time as they needed in order to understand the texts. Participants were then seated at the eye tracker and placed on a chin- and forehead-rest to minimize head movements. They then completed a calibration procedure. Before each trial, a fixation box appeared in the top left quadrant of the screen. Once the participant fixated this box, the texts would be presented. If the participants’ apparent point of fixation did not match with the fixation box, the experimenter recalibrated the eye tracker. Each trial consisted of one scenario, presented as four lines of text, with two blank lines between each line of text. Once the participants finished reading it, they looked away from the text and toward a post-it note affixed to the bottom right hand edge of the monitor, and then pressed the right-shoulder button on the console to progress to the next trial.

After 25% of the trials, a yes/no comprehension question appeared to ensure that the participant actually read and comprehended the text. The comprehension question (e.g., *“Were Dean and Chloe on holiday in Valencia?”*) related solely to the context of the scenario, and it was not a test of sarcasm comprehension. The average correct response rate of 94.7% indicates that participants were indeed reading for comprehension.

### Results and Discussion

Each scenario had three analysis regions. The critical region was the word that disambiguated the target utterance as being either sarcastic or literal. For example, in the scenario in Table 1, the critical word in the final comment *“Your suggestion is stirring!”* was *“stirring!”* The precritical region consisted of the two words preceding the critical region (e.g., *“suggestion is”*). The postcritical region was the remainder of the target utterance (e.g., *Dean said to her*.) Three measures of reading behavior are reported: *first-pass reading time* (the sum of all fixations in a region from first entering it until leaving it either via its left or right boundary, also known as *gaze duration* when the region comprises a single word), *regression path* (or *go-past*) reading time (the sum of all fixations from the time that a region is first entered until the region is left via its right region boundary), and *total reading time* (the sum of all fixations in a region, including fixations made when rereading the region).

Prior to the statistical analysis, the data were preprocessed using the EyeDoctor software (http://www.psych.umass.edu/eyelab). For each participant, the blinks were removed, and also the fixations were aligned on the vertical plane. The EyeDry software was then used to create the files needed for data analysis. Trials that had zero first-pass reading times for two consecutive regions (where regions were defined as a whole sentence in the context, the precritical, critical, and postcritical regions) were eliminated (discarded trials accounted for 2.6% of the data).

Data analysis was performed in R (R Core Team, 2013) using linear mixed effects modeling (*lme4* package) and potential interactions were decomposed in R using the function *testInteractions* from the *phia* package (where the chi-square is the default test; all reported *p* values are Bonferroni corrected). The first step was to discard 0-ms reading times from the analysis (see Table 2 for the percentage of data removed due to the reading time being 0 ms—typically due to participants skipping over the respective region). This was in addition to the 2.6% of the trials already removed due to having zero first-pass reading times for two consecutive regions.[Table-anchor tbl2]

The second step was to establish the appropriate random effects structure for each analysis. We started by fitting the maximal model to the data, as recommended by Barr, Levy, Scheepers, and Tily (2013). The random effects structure of the maximal model was: (1 + literality × explicitness|subject) + (1 + literality × explicitness|item). The reason why literality and explicitness were introduced as random slopes for both subjects and items is because both factors were within-subject, and within-item, respectively. However, because the maximal model failed to converge, the random effects structure had to be simplified in order to obtain convergence. This was done by progressively removing one random component at a time—the one that explained the least amount of variance in the previous nonconverging model.

The best way of establishing the appropriate random effects structure is currently a debatable issue. Barr et al. (2013) recommend always fitting the maximal model, with random slopes for all fixed effects of interest. However, this suggestion is often not practical—fitting the maximal model might often fail to converge because the model is overparameterized—the researcher is overfitting the data. The reason why Barr et al. (2013) argue for fitting the maximal model is because they claim that excluding a random slope for one of the fixed effects of interest increases the likelihood of making a Type I error. However, Matuschek and his colleagues have recently countered this point. They conducted a simulation study and concluded that the maximal model is not in fact the best choice, because although it reduces the likelihood of making a Type I error, it also significantly reduces power (Matuschek, Kliegl, Vasishth, Baayen, & Bates, 2015). Their study indicated that for factorial studies such as those reported in this article, it is recommended that the random effects structure of nonconverging models be reduced until a significant decrease in goodness of fit is observed. The model that is supported by the data (the one prior to a significant decrease in goodness of fit) provides the best balance between Type I error rates and power.

The analyses reported in this article reduce the random effects structure in a similar way to the procedure suggested in Matuschek et al. (2015), except that the simplification procedure is only continued until convergence is achieved rather than until a significant decrease in goodness of fit is observed (i.e., it is stopped earlier). That is because continuing until a significant decrease in goodness of fit is obtained leads to an even simpler random effects structure than the structure obtained by stopping the simplification process when a model converges. It is important to note that for the key findings reported in this article, both procedures lead to the same fixed-effects structures in the final models.

Once the random effects structure had been established, the third step was to perform a series of likelihood ratio tests comparing the fit of models with different fixed-effects structures in order to reach the best model fit for our data.^1^ The procedure used was to compare the model with the two factors in interaction with progressively simpler fixed-effects structures (that is, two main effects but no interaction, or only one main effect). See Table 3 for the models that had the best fit for our data and the values of their fixed-effects parameters. Furthermore, see Appendix D for the *t* values associated with the fixed factors that did not have significant effects (i.e., were not included in the best models), and the series of likelihood ratio tests performed in order to reach the best models.[Table-anchor tbl3]

#### The precritical region

**Figure 1 fig1:**
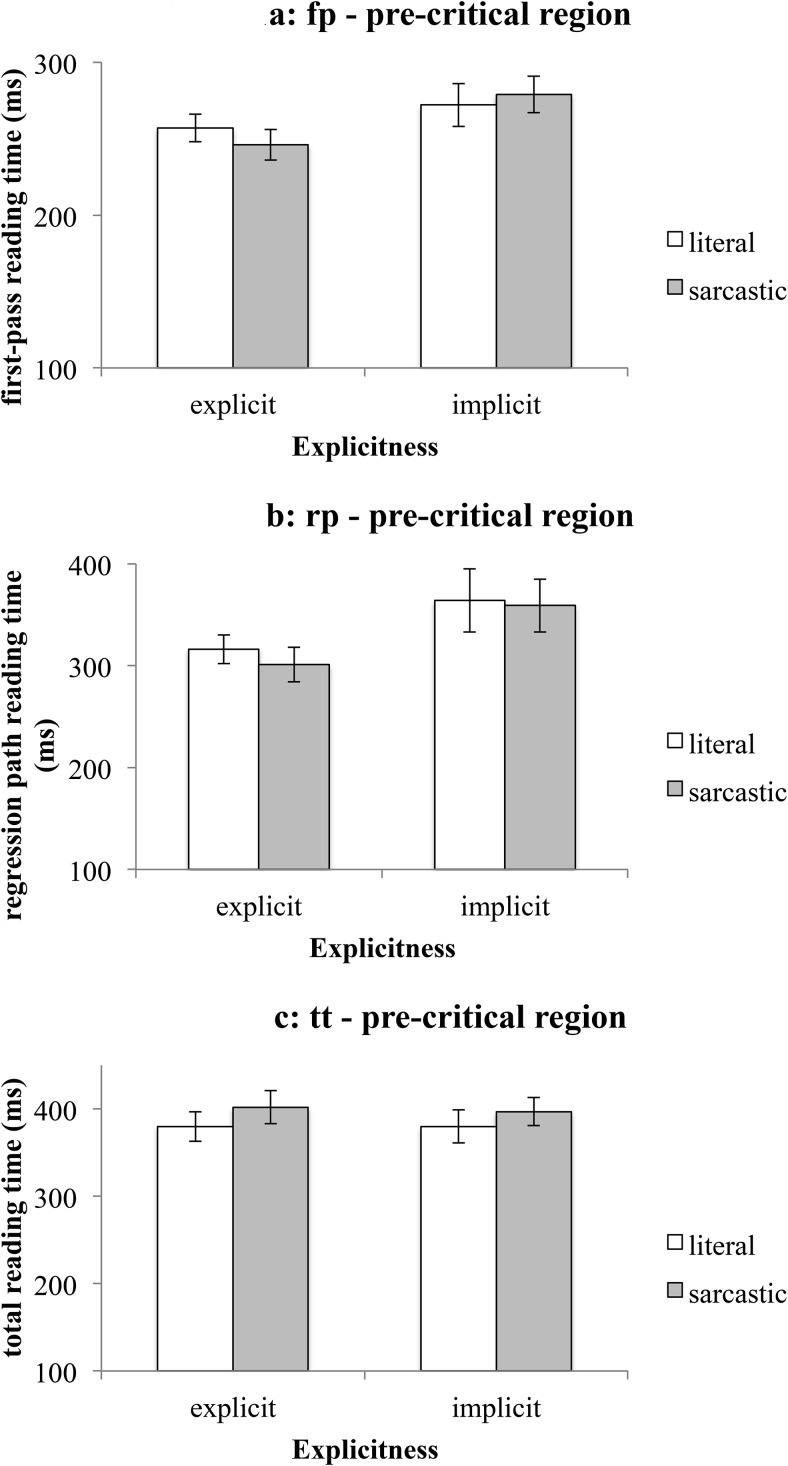
Mean reading times on the precritical region (Experiment 1). Figure 1a: first-pass reading time. Figure 1b: regression path reading time. Figure 1c: total reading time. Error bars represent ± 1*SEM*.

No effects were observed in first-pass or total reading times—see Figures 1a and 1c. However, regression path reading time was shorter following explicit contexts (*M*_rp-explicit_ = 309ms, *SEM* = 11 ms) than implicit ones (*M*_rp-implicit_ = 362 ms, *SEM* = 20 ms)—see Figure 1b. This suggests that even before reading the disambiguating word, participants reread the context in the implicit condition more than in the explicit one. Importantly, the null effect observed in first-pass reading time was reassuring in that it suggested that there was no baseline reading time difference between the experimental conditions.[Fig-anchor fig1]

#### The critical region

**Figure 2 fig2:**
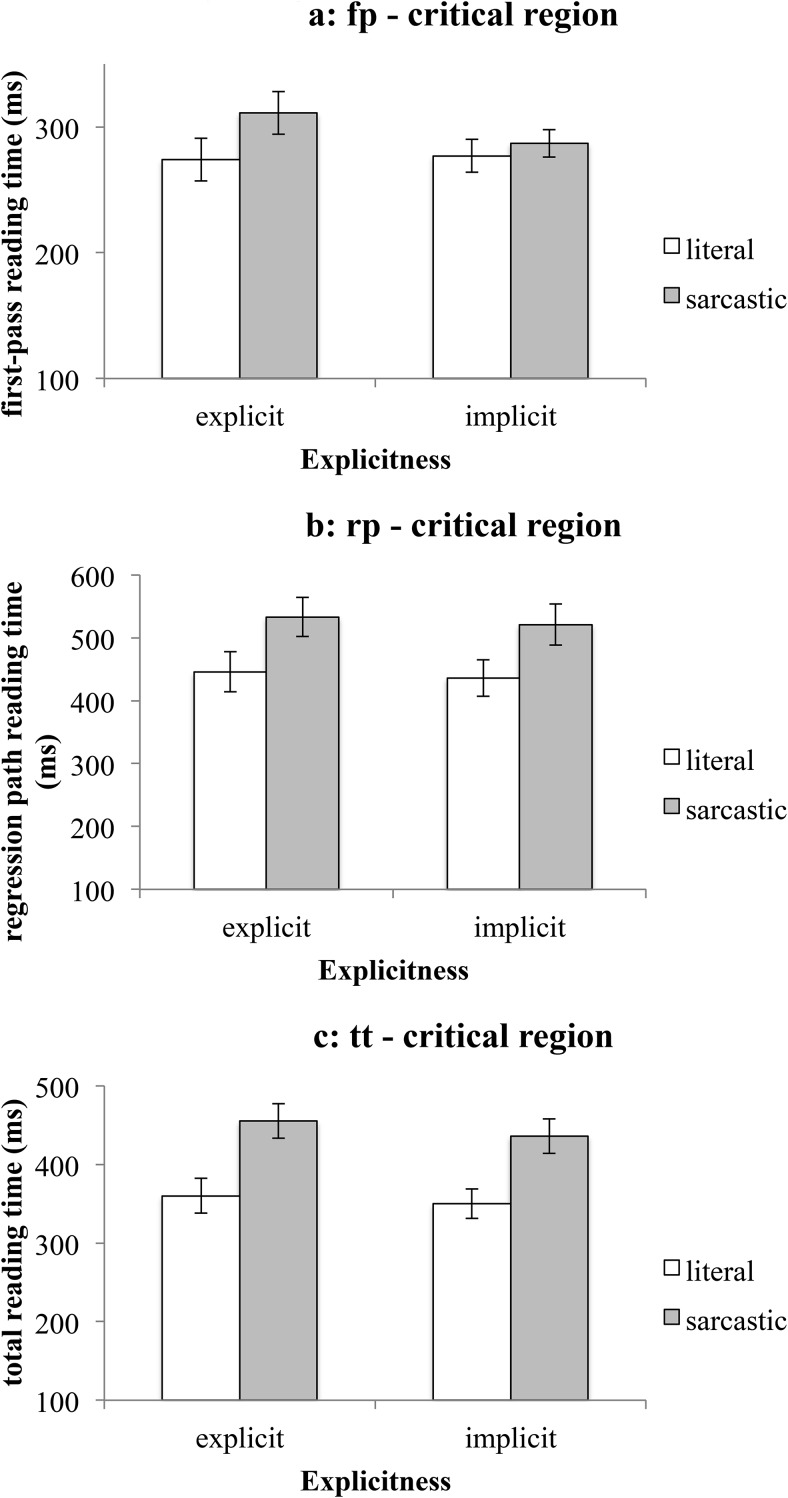
Mean reading times on the critical region (Experiment 1). Figure 2a: first-pass reading time. Figure 2b: regression path reading time. Figure 2c: total reading time. Error bars represent ± 1*SEM*.

There was a main effect of literality on all reading measures. Literal utterances were read faster (*M*_fp-literal_ = 275 ms, *SEM* = 11 ms; *M*_rp-literal_ = 441 ms, *SEM* = 22 ms; *M*_tt-literal_ = 355 ms, *SEM* = 15 ms) than sarcastic ones (*M*_fp-sarcastic_ = 299 ms, *SEM* = 10 ms; *M*_rp-sarcastic_ = 527 ms, *SEM* = 23 ms; *M*_tt-sarcastic_ = 446 ms, *SEM* = 15 ms)—see Figure 2a, 2b, and 2c below.[Fig-anchor fig2]

It seems that when the disambiguating word is encountered in the text, readers take longer to read it if it points toward a sarcastic interpretation of the comment, than if the comment’s intended meaning is literal. These results clearly support the predictions made by the modular accounts of sarcasm interpretation (the standard pragmatic model and the graded salience hypothesis), but offer no support for the implicit display theory’s prediction that sarcastic utterances in contexts containing an explicit expectation will be read as fast as literal utterances. In other words, it seems that we failed to support the prediction that increasing the degree of manifestness of the speaker’s expectation in the context offers an initial processing advantage for sarcastic utterances. These results are in line with those of previous studies of irony processing that report a literality effect (e.g., Filik et al., 2014 for unfamiliar ironies; Filik & Moxey, 2010; Giora, 1995; Giora et al., 1998, 2007; Kaakinen et al., 2014).

#### The postcritical region

**Figure 3 fig3:**
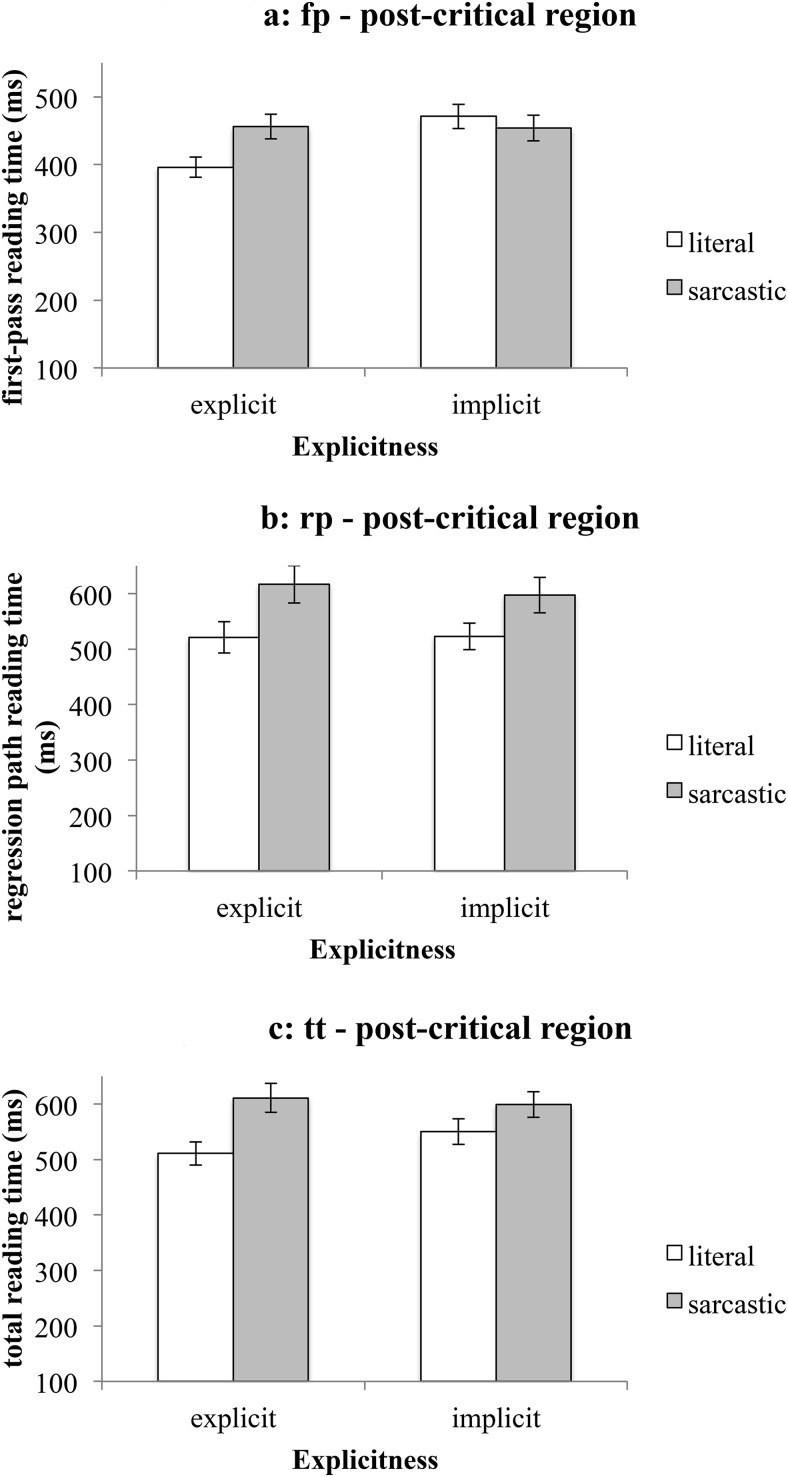
Mean reading times on the postcritical region (Experiment 1). Figure 3a: first-pass reading time. Figure 3b: regression path reading time. Figure 3c: total reading time. Error bars represent ± 1*SEM*.

An interaction between literality and explicitness was observed in first-pass reading time—see Figure 3a. Post hoc comparisons showed that (a) the region of text following a literal comment was read faster when the context was explicit (*M*_fp-literal-explicit_ = 396 ms, *SEM* = 15 ms) than when it was implicit (*M*_fp-literal-implicit_ = 471 ms, *SEM* = 18 ms): χ^*2*^(1, *N* = 32) = 8, *p* = .009, and (b) the region following a comment presented in an explicit context was read faster when the comment was literal (*M*_fp-literal-explicit_ = 396 ms, *SEM* = 15 ms) than when it was sarcastic (*M*_fp-sarcastic-explicit_ = 456 ms, *SEM* = 18 ms): χ^*2*^(1, *N* = 32) = 6.1, *p* = .027. Interestingly, this pattern of results was not due to sarcastic utterances becoming more difficult in implicit contexts, but due to literal utterances becoming more difficult in implicit contexts. We can conclude that the contextual manipulation seems to have an effect on the later stages of literal language processing, but not on the later stages of sarcasm processing.[Fig-anchor fig3]

Regression path and total reading times only reflected a main effect of literality—see Figure 3b and 3c. The region following a literal utterance was read faster (*M*_rp-literal_ = 522 ms, *SEM* = 18 ms; *M*_tt-literal_ = 531 ms, *SEM* = 15 ms) than following a sarcastic one (*M*_rp-sarcastic_ = 607 ms, *SEM* = 23 ms; *M*_tt-sarcastic_ = 605 ms, *SEM* = 17 ms). This pattern of results was also observed in Filik and Moxey’s (2010) study, and was taken to reflect difficulty in integrating the comment with the context when the comment is sarcastic. This difficulty in contextual integration seems to be independent of the explicitness of the speaker’s expectation in the context. Rather, as suggested by Filik and Moxey (2010), these results provide some evidence that after a sarcastic utterance is encountered, more reinspection of the text is required before the reader can comprehend the material, as compared with when a literal utterance is encountered, which is in line with the modular accounts of sarcasm comprehension (both the standard pragmatic model and the graded salience hypothesis), however, it fails to support the implicit display theory.

In conclusion, the results from Experiment 1 did not provide any support for the implicit display theory’s predictions that explicitness of the speaker’s expectation in the context would affect reading times for sarcastic utterances, by making them as easy to read as literal utterances when the expectation is explicit. However, it did provide support for both modular accounts’ predictions (the standard pragmatic model and the graded salience hypothesis), by showing that unfamiliar sarcastic utterances took longer to read than literal counterparts. In the next experiment we wanted to replicate the current results and additionally address the question of what role the properties of the utterance play in sarcasm comprehension. To this end, in Experiment 2 we investigated the online reading patterns of both familiar and unfamiliar sarcastic utterances presented in explicit and implicit contexts.

## Experiment 2

The aim of Experiment 2 was to replicate the results of Experiment 1, and extend them by investigating the role of the properties of the utterance in sarcasm comprehension, thus further discriminating between the predictions of the standard pragmatic model and the graded salience hypothesis. Hence, the factor of comment familiarity was added to the previous design. Under these circumstances, the two modular accounts would make different predictions for the time course of sarcasm comprehension. The standard pragmatic model would predict that sarcastic utterances would take longer to read under all circumstances, irrespective of comment familiarity or speaker’s expectation in the context. In other words, in order to support the standard pragmatic model, we would expect to find a main effect of literality, and no interactions with familiarity or explicitness. The graded salience hypothesis would predict that sarcastic utterances would take longer to read than literal counterparts if they were unfamiliar; however, if sarcastic utterances were familiar, they would be read as fast as literal counterparts. In other words, in order to support the graded salience hypothesis, we would expect to find an interaction between literality and familiarity, but no interaction with explicitness. The implicit display theory would predict that sarcastic utterances would take longer to read than literal ones if they are uttered in contexts in which the speaker’s expectation is implicit; however, if the speaker’s expectation is explicit, sarcastic utterances would be read as fast as literal ones. Thus, as in Experiment 1, we would expect to find an interaction between literality and explicitness, but no interaction with familiarity.

### Method

#### Participants

Sixty-four students from the University of Nottingham participated (*M*_age_ = 22 years and 6 months, *SD* = 7 months, 42 females and 22 males). None of them had taken part in Experiment 1. All participants were native English speakers, not diagnosed with any reading disorders, and had normal or corrected-to-normal vision. They either received a £4 inconvenience allowance for taking part, or course credit.

#### Materials and design

The experimental materials consisted of 48 short texts, with the same structure as the materials of the previous experiment (see Appendix E for a selection; the full set of items is available from the first author). The only difference in this experiment was that in the familiar condition the final utterance would be for example *“So excited!”* instead of *“Your suggestion is stirring!”* Thus the experiment consisted of a 2 literality (literal vs. sarcastic) × 2 speaker’s expectation (explicit vs. implicit) × 2 familiarity (familiar vs. unfamiliar) design, with literality and expectation as within-subject and within-item factors, and familiarity as a within-subject and between-items factor. The same set of stimulus norming tests were conducted as in Experiment 1, showing that (a) familiar comments were rated as significantly more familiar to the reader than the unfamiliar ones, (b) sarcastic comments were rated as significantly more sarcastic that the literal ones, (c) the expectation for how the other character should behave was significantly clearer in the explicit than in implicit conditions, (d) literal comments sounded more natural than sarcastic ones, and (e) the materials met the implicit display theory’s offline prediction: an expectation for sarcasm was significantly higher when the context was explicit compared with implicit (see Appendix F for the full set of results).

There were also 48 filler items, following a similar structure as in Experiment 1: half of the materials contained two characters but ended in a literal negative utterance, and the other half did not have any characters and were informative texts.

#### Procedure

The procedure was exactly the same as in Experiment 1. In terms of the comprehension questions, the average correct response rate was 93.9%, indicating again that participants read and correctly comprehended the scenarios.

### Results and Discussion

The scenarios in this experiment had the same three analysis regions as in Experiment 1 (i.e., *critical region*: disambiguating word, *precritical region*: two words prior to the disambiguating word, and *postcritical region*: the remainder of the target utterance). The data were preprocessed using the same software and procedures as before. Trials that had zero first-pass reading times for two consecutive regions were eliminated (removed trials accounted for 3.5% of the data).

Data analysis was performed in the same way as in Experiment 1. The first step was to discard 0-ms reading times from the analysis (see Table 4 for the percentage of data removed due to the reading time being 0 ms—typically due to participants skipping over the respective region). This was in addition to the 3.5% of the trials already removed due to having zero first-pass reading times for two consecutive regions. The second step was to establish the appropriate random effects structure, which was done following the same procedure as in Experiment 1. Once the random effects structure had been established, the third step was to perform a series of likelihood ratio tests comparing the fit of models with different fixed-effects structures in order to reach the best model fit for our data.^2^ The procedure used was to compare the model with the three factors in interaction with progressively simpler fixed-effects structures (that is, three models with two-way interactions and one main effect, followed by a model with three main effects, then three models with two main effects and finally three models with just one main effect). See Table 5 below for the models that had the best fit for our data and the values of their fixed-effects parameters. Furthermore, see Appendix G for the *t* values associated with the fixed factors that did not have significant effects (i.e., were not included in the best models), and the series of likelihood ratio tests performed in order to reach the best models.[Table-anchor tbl4][Table-anchor tbl5]

#### The precritical region

**Figure 4 fig4:**
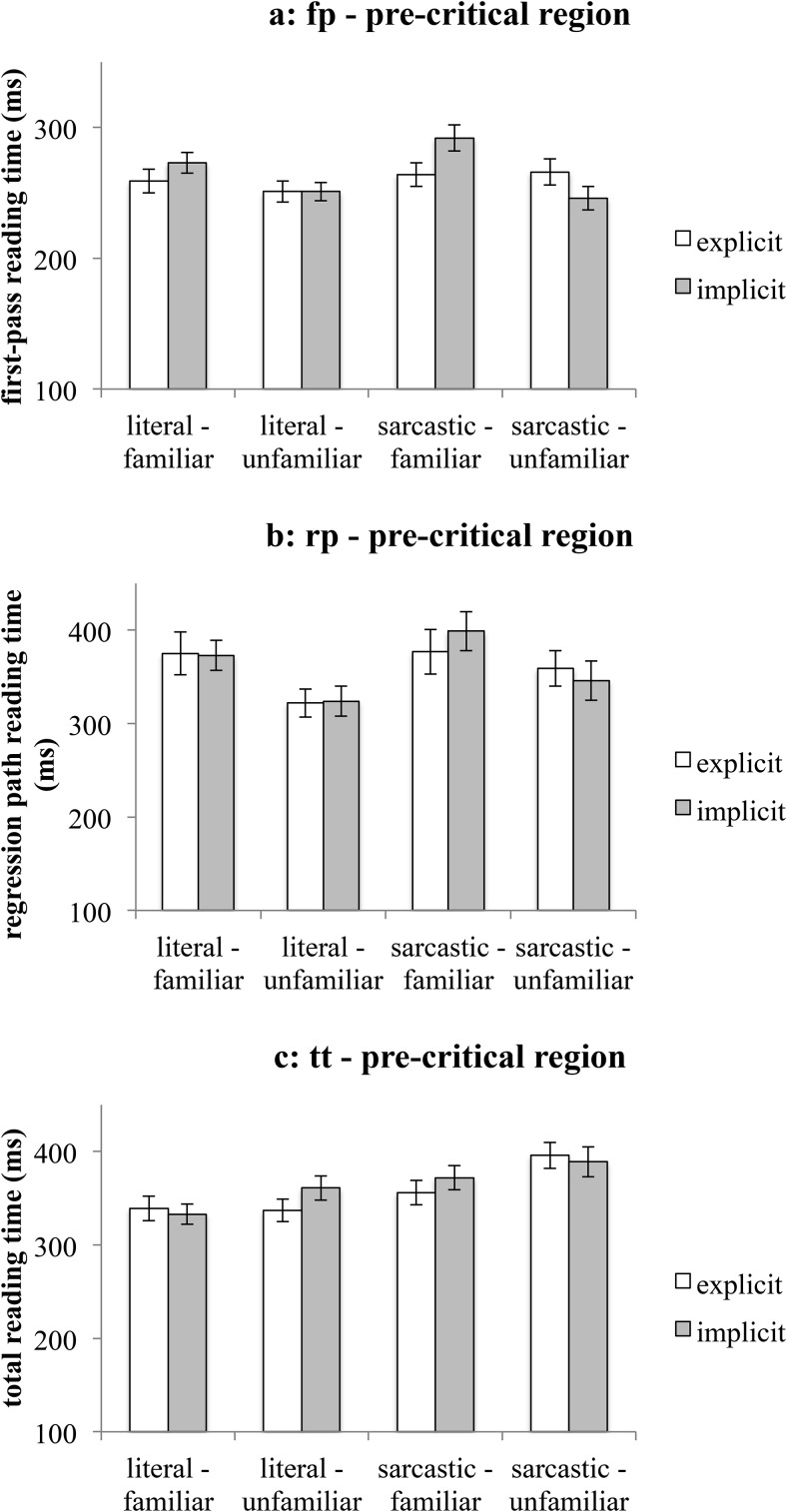
Mean reading times on the precritical region (Experiment 2). Figure 4a: first-pass reading time. Figure 4b: regression path reading time. Figure 4c: total reading time. Error bars represent ± 1*SEM*.

In first-pass reading time there was a familiarity-explicitness interaction—see Figure 4a. Post hoc tests indicated that (a) the precritical region of familiar comments was read faster if the context was explicit rather than implicit (*M*_fp-familiar-explicit_ = 262 ms, *SEM* = 6 ms, *M*_fp-familiar-implicit_ = 283 ms, *SEM* = 6 ms, χ^*2*^(1, *N* = 64) = 6.6, *p* = .02), but (b) the precritical region of unfamiliar comments were read in equal times in explicit and implicit contexts (*M*_fp-unfamiliar-explicit_ = 258 ms, *SEM* = 6 ms, *M*_fp-unfamiliar-implicit_ = 248 ms, *SEM* = 6 ms, χ^*2*^(1, *N* = 64) = 1.4, *p* = .5). This indicates that even before the readers knew whether the comment was going to be literal or sarcastic, the context had an impact on the reading times of familiar comments, but not on the unfamiliar ones.[Fig-anchor fig4]

In regression path reading time, the familiarity effect indicated that the precritical region of familiar utterances was read slower than that of unfamiliar ones—see Figure 4b. However, because this specific comparison is between reading times on different words, any simple main effects of familiarity are very difficult to interpret meaningfully.

In total reading times, the literality main effect indicated that the precritical region of literal comments was read faster (*M*_tt-literal_ = 343 ms, *SEM* = 6 ms) than that of sarcastic comments (*M*_tt-sarcastic_ = 378 ms, *SEM* = 7 ms) – see Figure 4c. The most likely interpretation of the literality main effect is that the precritical region has been reread more in sarcastic scenarios than in literal ones, which might suggest a difficulty in the interpretation of the sarcastic materials as predicted by the standard pragmatic model. The familiarity main effect indicated that the precritical region was read faster in familiar than unfamiliar utterances, but as explained above, the familiarity main effect alone cannot be interpreted meaningfully.

#### The critical region

**Figure 5 fig5:**
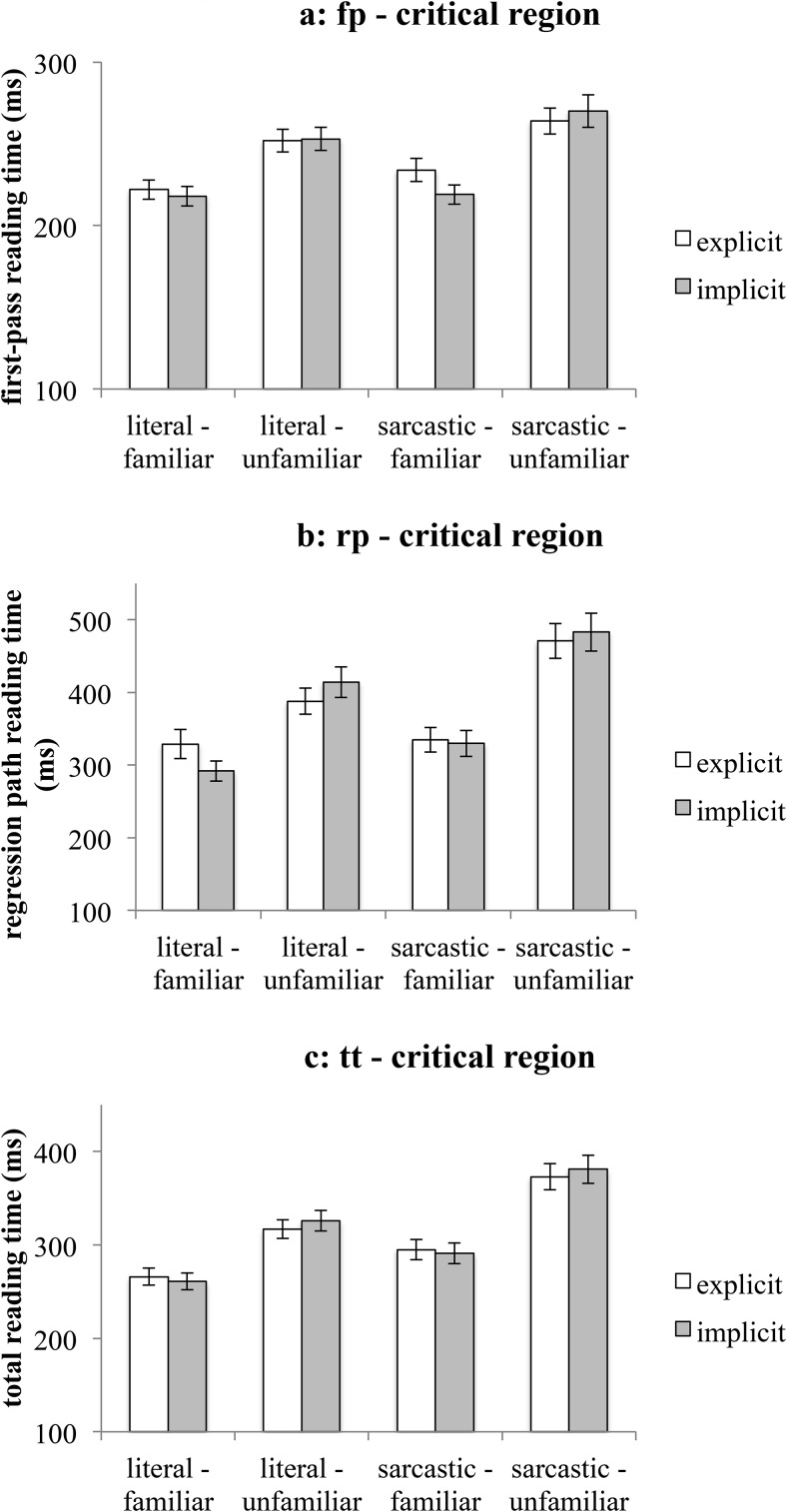
Mean reading times on the critical region (Experiment 2). Figure 5a: first-pass reading time. Figure 5b: regression path reading time. Figure 5c: total reading time. Error bars represent ± 1*SEM*.

In first-pass reading time, there were two main effects—see Figure 5a. The critical word of familiar utterances was read faster (*M*_fp-familiar_ = 223 ms, *SEM* = 3 ms) than the critical word of unfamiliar utterances (*M*_fp-unfamiliar_ = 260 ms, *SEM* = 4 ms). Again, although this result is in the direction that one might expect, it should be interpreted with caution, because this specific comparison is between reading times on different words (e.g., *excited* in the familiar condition vs. *stirring* in the unfamiliar condition). The literality main effect indicated that the critical word of a literal comment was read faster (*M*_fp-literal_ = 237 ms, *SEM* = 3 ms) than that of a sarcastic comment (*M*_fp-sarcastic_ = 248 ms, *SEM* = 4 ms). This pattern of results supports the predictions of the standard pragmatic model, indicating that in the early processing stages, sarcasm seems to indeed be overall more difficult to process than literal language, irrespective of familiarity or contextual information.[Fig-anchor fig5]

In regression path reading time, an interaction was observed between literality and familiarity - see Figure 5b. Post hoc comparisons showed that (a) unfamiliar utterances were slower to read in the sarcastic condition than in the literal condition (*M*_rp-unfamiliar-literal_ = 402 ms, *SEM* = 14 ms, *M*_rp-unfamiliar-sarcastic_ = 477 ms, *SEM* = 17 ms): χ^*2*^(1, *N* = 64) = 15.7, *p* < .001, and (b) familiar utterances were read equally fast irrespective of whether they were sarcastic or literal (*M*_rp-familiar-literal_ = 311 ms, *SEM* = 12 ms, *M*_rp-familiar-sarcastic_ = 333 ms, *SEM* = 12 ms): χ^*2*^(1, *N* = 64) = 0.8, *p* = .8. This pattern of results fully supports the graded salience hypothesis, but offers no support for the standard pragmatic model or the implicit display theory. Sarcastic utterances do not always take longer to read than literal ones (as the standard pragmatic model would predict), and there is currently no evidence that they are influenced by the strength of contextual information (as the implicit display theory would predict). However, when they are familiar, sarcastic utterances are read as fast as literal utterances, as predicted by the graded salience hypothesis.

Finally in total reading time, an interaction between literality and familiarity was observed again—see Figure 5c. However, this time, literal comments were read faster than sarcastic ones in both familiar (*M*_tt-familiar-literal_ = 264 ms, *SEM* = 6 ms, *M*_tt-familiar-sarcastic_ = 293 ms, *SEM* = 8 ms, χ^*2*^(1, *N* = 64) = 5.9, *p* = .03) and unfamiliar conditions (*M*_tt-unfamiliar-literal_ = 321 ms, *SEM* = 8 ms, *M*_tt-unfamiliar-sarcastic_ = 377 ms, *SEM* = 10 ms, χ^*2*^(1, *N* = 64) = 22.3, *p* < .001). This result suggested that the advantage for familiar sarcastic comments did not carry over into the later stages of processing, and instead both familiar and unfamiliar sarcastic comments seemed to require additional processing time compared with literal their counterparts. In line with the findings from Experiment 1, these results also fail to support the implicit display theory, because the explicitness of the speaker’s expectation did not facilitate sarcasm processing in any of the conditions.

#### The postcritical region

**Figure 6 fig6:**
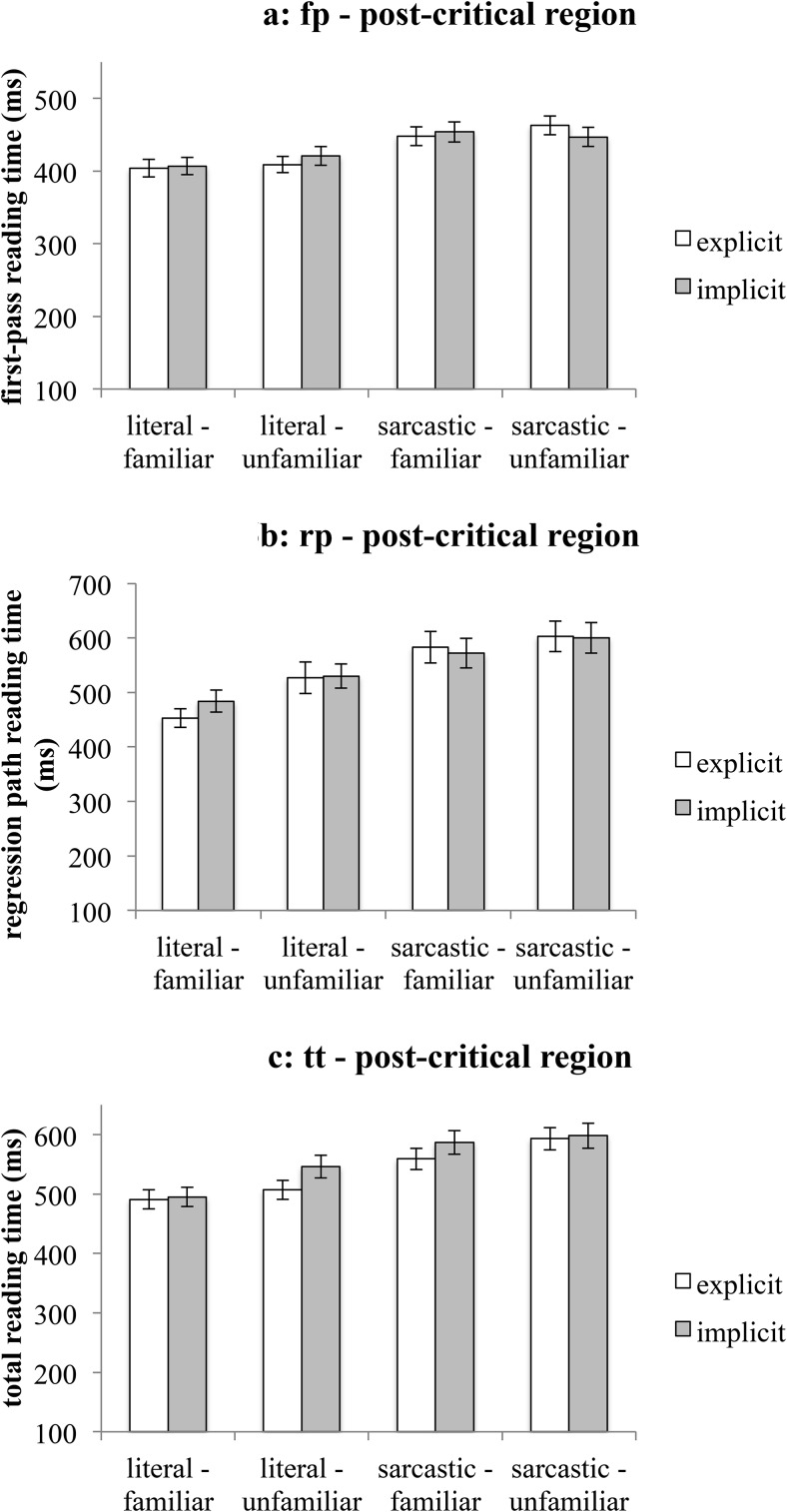
Mean reading times on the postcritical region (Experiment 2). Figure 6a: first-pass reading time. Figure 6b: regression path reading time. Figure 6c: total reading time. Error bars represent ± 1*SEM*.

In first-pass reading time, a main effect of literality was observed—see Figure 6a. The region of text following a literal utterance had shorter first-pass reading times (*M*_fp-literal_ = 411 ms, *SEM* = 6 ms) than the region following a sarcastic utterance (*M*_fp-sarcastic_ = 453 ms, *SEM* = 7 ms). In regression path reading times and total reading times, two main effects of literality and familiarity were observed—see Figure 6b and 6c. The region of text following a literal utterance was read faster (*M*_rp-literal_ = 499 ms, *SEM* = 11 ms; *M*_tt-literal_ = 510 ms, *SEM* = 8 ms) than the region following a sarcastic utterance (*M*_rp-sarcastic_ = 590 ms, *SEM* = 14 ms; *M*_tt-sarcastic_ = 584 ms, *SEM* = 10 ms). Also the region following a familiar utterance was read faster (*M*_rp-familiar_ = 524 ms, *SEM* = 12 ms; *M*_tt-familiar_ = 533 ms, *SEM* = 9 ms) than the region following an unfamiliar utterance (*M*_rp-unfamiliar_ = 565 ms, *SEM* = 13 ms; *M*_tt-unfamiliar_ = 561 ms, *SEM* = 9 ms). These results seem to support the findings from Experiment 1 and those observed in Filik and Moxey’s (2010) study, which showed that the region of text following sarcastic utterances is read more slowly than the text following literal utterances.[Fig-anchor fig6]

The current experiment showed that although familiarity offers an advantage for the processing of familiar sarcastic utterances when they are initially encountered (as evidenced in regression path reading times on the disambiguating word), this advantage is lost in the later stages of processing (as illustrated by the lack of an interaction between literality and familiarity on the postcritical region).

## General Discussion

Two experiments were carried out to contribute to the current theoretical debate on the factors affecting sarcasm processing, using tightly controlled materials, and a method (eye-tracking) sensitive enough to reveal both early and late effects of our manipulations. In both experiments, participants read short scenarios while their eye movements were recorded. In Experiment 1, the contexts of these scenarios either included an explicit expectation of the speaker, or an implicit expectation, and they ended in either a literal comment or an unfamiliar sarcastic one. This design was used in order to test the conditions under which the predictions of the modular accounts and the implicit display theory differ the most. In Experiment 2 the familiarity of the sarcastic comment was manipulated, in addition to explicitness of expectation, in order to also assess the role of certain properties of the utterance itself in sarcasm comprehension.

### The Early Stages of Sarcasm Processing

In the two experiments reported here, initial processing was considered to be reflected in the reading times of the critical word before participants moved on to the next text region (that is, first-pass and regression path reading times on the disambiguating word). For this critical disambiguating region, we found that unfamiliar sarcastic utterances took longer to read than literal utterances (Experiments 1 and 2), but familiar sarcastic utterances were read as quickly as literal ones (regression path reading times in Experiment 2).

The results of both experiments are most in line with the predictions made by the graded salience hypothesis, and less so with those of the standard pragmatic model or the implicit display theory. The familiarity of the target comments (rather than context or their literality) seems to have an influence on the initial processing of sarcasm comprehension, in the direction predicted by the graded salience hypothesis. In the early stages of processing, when sarcastic utterances were familiar, they were read as fast as literal utterances, while unfamiliar sarcastic utterances were read more slowly than their literal counterparts. These results are in line with those of Giora and her colleagues (1995, 1998, 2007), Filik and Moxey (2010); Filik et al. (2014, Experiment 1), and Frisson and Pickering (1999). Furthermore, they are also in line with those of Filik et al.’s (2014) ERP study of irony processing. Filik et al. found that in the N400 time range, the ERP amplitudes were modulated by the literality and familiarity of the comment, in that unfamiliar ironies had more negative-going amplitudes compared with literal items, whereas an amplitude difference was not observed between familiar sarcastic and literal utterances. This pattern of results nicely mirrors our eye-tracking results for regression path reading times on the critical region (Experiment 2), and stands to show that when readers encounter the disambiguating word of an unfamiliar irony, they take longer to read it, which seems to be because of semantic difficulties associated with processing its meaning (as reflected in the ERP). However, this semantic integration difficulty is not observed for the disambiguating word of familiar ironies.

There were no main effects of the explicitness of the speaker’s expectation, nor interactions of this factor with literality or familiarity in the critical region (Experiments 1 and 2). These results do not support the prediction of the implicit display theory that when the degree of manifestness of an expectation in the context is high, sarcastic utterances would be read as fast as literal ones, even though we have offline evidence that a sarcastic utterance was expected more when the context explicitly mentioned the speaker’s expectation. The two experiments reported in this article seem to suggest that making a speaker’s expectation explicit in the context did not facilitate comprehension of sarcasm. However, because a role for context has been reported previously in the literature, we believe that further research is needed to clarify whether contexts with even more explicit expectations than those employed in our study would elicit a functional effect on sarcasm comprehension. Although we ensured that the difference between the explicit and implicit conditions was statistically significant, it is difficult to say just how explicit the speaker’s expectation needs to be in order for us to observe a functional effect.

### The Later Stages of Sarcasm Processing

In the two experiments reported here, we assume that later stages of processing are reflected in measures of reading time after the participants have first processed the critical region (that is, in the total reading times on the precritical and critical region, and all reading measures on the postcritical region).

Even though, as noted before, an interaction between literality and familiarity was observed in the early reading measures on the critical region in Experiment 2, this interaction was no longer observed in the later reading stages, that is, familiar sarcastic utterances lost their advantage and became more difficult to process than literal ones. This reading pattern suggests that although familiar sarcastic utterances have an initial advantage, they still give rise to processing difficulties after the first reading, when participants reread the disambiguating word. Therefore, in the later stages of processing, sarcasm comprehension seems to have an additional processing cost compared with literal language comprehension.

Our finding that familiarity effects disappear in the later stages of sarcasm comprehension is in line with the results of Filik et al.’s (2014) ERP experiment. They found that in the P600 time range, the ERP amplitudes were only modulated by literality (and not familiarity), with ironies showing more positive-going amplitudes than literal utterances. De Grauwe, Swain, Holcomb, Ditman, and Kuperberg’s (2010) ERP study of metaphor also points toward this conclusion. Their study involved participants reading sentences that contained either a familiar metaphor, or was simply a literal clause. The results showed a P600 effect for metaphors as compared with literal sentences, which was interpreted as a reflection of the difficulties associated with the integration of figurative utterances with the context.

An explanation provided for their result is that in the later stages of comprehension, both the literal and figurative meanings are activated. For sarcasm, it would mean that in the later stages of processing there is an ongoing conflict between the literal and ironic meanings of the sarcastic utterances, which is not affected by the familiarity of the utterance. This conclusion is supported by the indirect negation view proposed by Giora (1995), which predicts that both the literal and ironic meanings of a sarcastic utterance are retained in the later stages of processing in order for the difference between them to be computed.

With regards to the late effects of our contextual manipulation, we found no evidence that making the speaker’s expectation explicit in the context facilitates sarcasm comprehension. However, we did observe some evidence that the contextual manipulation affected the reading time of literal utterances, which became more difficult to process in implicit contexts (see first-pass reading times on the postcritical region, Experiment 1). Therefore it is not the case that our contextual manipulation did not have any effects, it is only the case that it did not affect sarcasm processing in the way predicted by the implicit display theory.

The finding that sarcasm comprehension is overall more difficult than literal language comprehension in the later stages of processing could potentially be compatible with the predictions of the standard pragmatic model, because according to this theory, readers or listeners need to reanalyze the sarcastic materials before making a correct interpretation, which would result in a processing cost. However, the standard pragmatic model cannot explain the early processing advantage of familiar sarcastic utterances as compared with literal ones. On the other hand, the graded salience hypothesis predicts the early ease of processing of sarcastic utterances that we observed, and can also explain our findings for the later stages of processing in terms of a conflict between the two meanings of a sarcastic utterance (as explained above). Therefore, it seems that out of the two modular accounts discussed in this article, our results are more compatible with the graded salience hypothesis than the standard pragmatic model.

The present results could also potentially be explained by the constraint satisfaction model (Pexman, 2008). As described in the Introduction, this framework theory allows for many unspecified factors to affect sarcasm comprehension, and thus does not make clear predictions about any specific factors. However, the constraint satisfaction model could be used to frame our results, and we could potentially now specify that one factor that affects sarcasm comprehension is the comment’s familiarity, but that we do not have any evidence yet that the speaker’s expectation is also a factor.

In conclusion, by using experimental designs that could fully test the role of sentence familiarity, and of contextual factors (explicitness of speaker’s expectation) in sarcasm comprehension, and by employing a sensitive and ecologically valid online methodology, the results from the two experiments reported in this article offer more support for the graded salience hypothesis than the standard pragmatic model or the implicit display theory. The familiarity of the meaning of a sarcastic utterance seems to influence its processing time, making it as easy to read as literal utterances in the early stages of processing. However, this beneficial effect does not seem to carry over to later stages of processing, when sarcastic utterances take longer to process than literal utterances, irrespective of degree of familiarity. These results are best explained by the graded salience hypothesis and the indirect negation view, because they seem to suggest that the familiarity of a comment is an important factor in sarcasm processing, and that both the literal and sarcastic meanings of a sarcastic comment may be retained for further processing in the later stages of comprehension. We failed to support the prediction of the implicit display theory that making the speaker’s expectation explicit in the context would provide support for sarcasm comprehension (see also Giora et al., 2009). However, just because this specific factor did not have a visible functional effect on processing, does not mean that other factors proposed by the implicit display theory could not have one. This remains a question for future studies to address. One potential goal for future studies could be to specify which contextual factors do affect the time course of sarcasm comprehension, and transform the parallel constraint satisfaction model from a framework theory, into a theory with clear testable predictions.

## Figures and Tables

**Table 1 tbl1:** Example Scenario (Experiment 1)

Literality	Explicitness	Example scenario
Literal	Explicit	Dean and Chloe were on holiday in Valencia for a week. The end of the trip was approaching so Dean asked Chloe to think of something thrilling to do on their last day. Chloe suggested they go and watch the Formula 1 race, which was Dean’s favourite sport. “Your/ suggestion is precritical region/ stirring!” critical region/ Dean said to her. Postcritical region/ They went out.
	Implicit	Dean and Chloe were on holiday in Valencia for a week. Their trip was quickly coming to an end, and they weren’t sure what to do on their final day. Chloe suggested they go and watch the Formula 1 race, which was Dean’s favourite sport. “Your/ suggestion is precritical region/ stirring!” critical region/ Dean said to her. Postcritical region/ They went out.
Sarcastic	Explicit	Dean and Chloe were on holiday in Valencia for a week. The end of the trip was approaching so Dean asked Chloe to think of something thrilling to do on their last day. Chloe suggested they stay in the hotel and watch TV, which was quite boring. “Your/ suggestion is precritical region/ stirring!” critical region/ Dean said to her. Postcritical region/ They went out.
	Implicit	Dean and Chloe were on holiday in Valencia for a week. Their trip was quickly coming to an end, and they weren’t sure what to do on their final day. Chloe suggested they stay in the hotel and watch TV, which was quite boring. “Your/ suggestion is precritical region/ stirring!” critical region/ Dean said to her. Postcritical region/ They went out.

**Table 2 tbl2:** Summary of 0-ms Reading Time Removal (Experiment 1)

Analysis region	Reading measure	% of missing data
Precritical	fp	17.4
	rp	17.4
	tt	7.2
Critical	fp	11.6
	rp	11.6
	tt	8.4
Postcritical	fp	0.5
	rp	0.5
	tt	0.1
*Note.* fp = first-pass; rp = regression path; tt = total reading time.		

**Table 3 tbl3:** Best Fitting Models and Fixed-Effects Parameters (Experiment 1)

Analysis region	Reading measure	Model	Fixed effects	Coefficient	*SE*	*t*
Precritical	fp	∼ 1 + (1 + explicitness | subject) + (1 + explicitness | item)	(Intercept)	252.5	10	25
	rp	∼ explicitness + (1 | subject) + (1 | item)	(Intercept)	307.9	21	14.6
			Explicitness	54.4	22.3	2.4
	tt	∼ 1 + (1 + literality × explicitness | subject) + (1 + literality × explicitness | item)	(Intercept)	383.4	23.4	16.4
Critical	fp	∼ 1 + (1 + literality × explicitness | subject) + (1 + literality × explicitness | item)	(Intercept)	276.2	16.6	16.6
	rp	∼ literality + (1 | subject) + (1 + explicitness | item)	(Intercept)	424	45.4	9.3
			Literality	92	25.7	3.6
	tt	∼ literality + (1 + literality | subject) + (1 + literality × explicitness | item)	(Intercept)	343.5	32.2	10.7
			Literality	87.9	23	3.8
Postcritical	fp	∼ literality × explicitness + (1 + literality × explicitness | subject) + (1 + literality × explicitness | item)	(Intercept)	394.3	21.5	18.4
			Literality	66.1	26.7	2.5
			Explicitness	78.5	27.8	2.8
			Literality × explicitness	−87.3	35.5	−2.5
	rp	∼ literality + (1 + literality × explicitness | subject) + (1 + literality × explicitness | item)	(Intercept)	530.2	35.3	15.02
			Literality	80.5	30	2.7
	tt	∼ literality + (1 + literality × explicitness | subject) + (1 + literality × explicitness | item)	(Intercept)	518.3	33	15.7
			Literality	66.1	23.3	2.8
*Note.* fp = first-pass; rp = regression path; tt = total reading time.						

**Table 4 tbl4:** Summary of 0-ms Reading Time Removal (Experiment 2)

Analysis region	Reading measure	% of missing data
Precritical	fp	17.1
	rp	17.1
	tt	8.2
Critical	fp	17
	rp	17
	tt	14
Postcritical	fp	1.9
	rp	1.9
	tt	1
*Note.* fp = first-pass; rp = regression path; tt = total reading time.		

**Table 5 tbl5:** Best Fitting Models and Fixed-Effects Parameters (Experiment 2)

Analysis region	Reading measure	Model	Fixed effects	Coefficient	*SE*	*t*
Precritical	fp	∼ familiarity × explicitness + (1 + literality | subject) + (1 | item)	(Intercept)	257.9	9.6	26.9
			Familiarity	−3.3	8	−0.4
			Explicitness	20.3	7.9	2.6
			Familiarity × explicitness	−29.6	11.2	−2.6
	rp	∼ familiarity + (1 + literality | subject) + (1 | item)	(Intercept)	375.9	17	22.2
			Familiarity	−42.1	13.3	−3.2
	tt	∼ literality + familiarity + (1 + literality × explicitness | subject) + (1 | item)	(Intercept)	328.5	15.9	20.7
			Literality	37.9	9	4.2
			Familiarity	23	8	2.9
Critical	fp	∼ literality + familiarity + (1 + explicitness | subject) + (1 + literality | item)	(Intercept)	212.2	8	26.5
			Literality	10.3	4.9	2.1
			Familiarity	38.9	4.6	8.5
	rp	∼ literality × familiarity + (1 + explicitness | subject) + (1 + literality | item)	(Intercept)	307.3	20.8	14.7
			Literality	17.6	20.2	0.9
			Familiarity	92.4	19	4.9
			Literality × Familiarity	57.9	26.8	2.2
	tt	∼ literality × familiarity + (1 + literality × familiarity | subject) + (1 | item)	(Intercept)	258.7	13.2	19.6
			Literality	26.5	10.9	2.4
			Familiarity	59.3	10.6	5.6
			Literality × familiarity	29.1	14.6	2
Postcritical	fp	∼ literality + (1 + literality × explicitness | subject) + (1 | item)	(Intercept)	408.9	17.2	23.8
			Literality	42.5	7.8	5.5
	rp	∼ literality + familiarity + (1 + literality × familiarity | subject) + (1 | item)	(Intercept)	470.7	23.9	19.7
			Literality	90.4	22.5	4
			Familiarity	46.7	17.7	2.6
	tt	∼ literality + familiarity + (1 + literality × explicitness | subject) + (1 + literality | item)	(Intercept)	490.6	25.4	19.3
			Literality	74.2	18.6	4
			Familiarity	31.6	10.5	3
*Note.* fp = first-pass; rp = regression path; tt = total reading time.						

**Table D1 tbl6:** Table for the t Values of the Nonsignificant Fixed Effects

Analysis region	Reading measure	Fixed effects (from full model)	*t*
Precritical	fp	Literality	−0.6
		Explicitness	0.9
		Literality × explicitness	0.9
	rp	Literality	−0.4
		Literality × explicitness	0.2
	tt	Literality	0.8
		Explicitness	0.2
		Literality × explicitness	−0.1
Critical	fp	Literality	1.8
		Explicitness	.001
		Literality × explicitness	−0.8
	rp	Explicitness	−0.2
		Literality × explicitness	−0.4
	tt	Explicitness	−0.6
		Literality × explicitness	−0.4
Postcritical	fp		
	rp	Explicitness	−0.03
		Literality × explicitness	−0.4
	tt	Explicitness	1.3
		Literality × explicitness	−1.4
*Note*. As a rule of thumb, only effects with |t| > 2 are likely to be significant (Baayen, Davidson, & Bates, 2008).			

**Table D2 tbl7:** Series of Likelihood Ratio Tests, Their AIC, and p Values (Experiment 1)

Model number	Fixed-effects structure	AIC	*p* (vs. Model no.)
fp—precritical			
1	Literality × explicitness	7,775	
2	Literality + explicitness	7,774	.4 (vs. 1)
3	Literality	7,775	.077 (vs. 2)
4	Explicitness	7,772	.9 (vs. 2)
**5**	**Intercept**	**7,773**	**.9 (vs. 3) .07 (vs. 4)**
rp—precritical			
1	Literality × explicitness	8,638	
2	Literality + explicitness	8,636	.8 (vs. 1)
3	Literality	8,640	.015 (vs. 2)
**4**	**Explicitness**	**8,635**	**.7 (vs. 2)**
5	Intercept	8,639	.015 (vs. 4)
tt—precritical			
1	Literality × explicitness	9339	
2	Literality + explicitness	9,337	.9 (vs. 1)
3	Literality	9,335	.8 (vs. 2)
4	Explicitness	9,336	.3 (vs. 2)
**5**	**Intercept**	**9,334**	**.3 (vs. 3) .7 (vs. 4)**
fp—critical			
1	Literality × explicitness	8,643	
2	Literality + explicitness	8,642	.4 (vs. 1)
3	Literality	8,641	.4 (vs. 2)
4	Explicitness	8,643	.078 (vs. 2)
**5**	**Intercept**	**8,642**	**.062 (vs. 3) .3 (vs. 4)**
rp—critical			
1	Literality × explicitness	9,555	
2	Literality + explicitness	9,553	.7 (vs. 1)
**3**	**Literality**	**9,551**	**.6 (vs. 2)**
4	Explicitness	9,563	<.001 (vs. 2)
5	Intercept	9,562	<.001 (vs. 3)
tt—critical			
1	Literality × explicitness	9,372	
2	Literality + explicitness	9,370	.7 (vs. 1)
**3**	**Literality**	**9,370**	**.3 (vs. 2)**
4	Explicitness	9,380	<.001 (vs. 2)
5	Intercept	9,378	<.001 (vs. 3)
fp—postcritical			
**1**	**Literality** × **explicitness**	**9,990**	
2	Literality + explicitness	9,994	.019 (vs. 1)
rp—postcritical			
1	Literality × explicitness	10,852	
2	Literality + explicitness	10,850	.7 (vs. 1)
**3**	**Literality**	**10,848**	**.6 (vs. 2)**
4	Explicitness	10,854	.01 (vs. 2)
5	Intercept	10,852	.01 (vs. 3)
tt—postcritical			
1	Literality × explicitness	10,408	
2	Literality + explicitness	10,408	.2 (vs. 1)
**3**	**Literality**	**10,406**	**.7 (vs. 2)**
4	Explicitness	10,413	.01 (vs. 2)
5	Intercept	10,411	.01 (vs. 3)
*Note.* In this table, the fixed-effects structure gets progressively simpler at every step; a *p* value < .05 suggests that the better model fit is the one with the more complex fixed-effects structure out of the two models being compared; similarly, a *p* value > .05 suggests that it is the simpler fixed-effects structure that best describes the data. The fixed-effects structure of the best model fit is in boldface. AIC = Akaike’s information criterion (the smaller the AIC, the better the model fit; Wagenmakers & Farrell, 2004).			

**Table G1 tbl8:** Series of Likelihood Ratio Tests, Their AIC, and p Values (Experiment 1)

Analysis region	Reading measure	Fixed effects (from full model)	*t*
Precritical	fp	Literality	.5
		Literality × explicitness	1
		Literality × familiarity	.6
		Literality × familiarity × explicitness	−1.6
	rp	Literality	.1
		Explicitness	−.1
		Literality × explicitness	.8
		Literality × familiarity	.8
		Explicitness × familiarity	.2
		Literality × familiarity × explicitness	−.9
	tt	Explicitness	−.4
		Literality × explicitness	.9
		Literality × familiarity	1.9
		Explicitness × familiarity	1.4
		Literality × familiarity × explicitness	−1.8
Critical	fp	Explicitness	−.3
		Literality × explicitness	−.9
		Literality × familiarity	−.04
		Explicitness × familiarity	.2
		Literality × familiarity × explicitness	1
	rp	Explicitness147–2	−1.2
		Literality × explicitness	.8
		Explicitness × familiarity	1.5
		Literality × familiarity × explicitness	−.8
	tt	Explicitness	−.4
		Literality × explicitness	−.03
		Explicitness × familiarity	.5
		Literality × familiarity × explicitness	−.03
Postcritical	fp	Explicitness	−.2
		Familiarity	.4
		Literality × explicitness	.2
		Literality × familiarity	.5
		Explicitness × familiarity	.5
		Literality × familiarity × explicitness	−1
	rp	Explicitness	.9
		Literality × explicitness	−1
		Literality × familiarity	−1
		Explicitness × familiarity	−.7
		Literality × familiarity × explicitness	.6
	tt	Explicitness	.05
		Literality × explicitness	.7
		Literality × familiarity	.5
		Explicitness × familiarity	1.1
		Literality × familiarity × explicitness	−1.2
*Note.* As a rule of thumb, only effects with |t| > 2 are likely to be significant (Baayen, Davidson, & Bates, 2008).			

**Table G2 tbl9:** Series of Likelihood Ratio Tests, Their AIC, and *p* Values (Experiment 2)

Model number	Fixed-effects structure	AIC	*p* (vs. model no.)
fp—precritical			
1	Literality × explicitness × familiarity	31,202	
2	Literality × familiarity + explicitness	31,206	.02 (vs. 1)
3	Literality × explicitness + familiarity	31,207	.015 (vs. 1)
4	Familiarity × explicitness + literality	31,200	.3 (vs. 1)
**5**	**Familiarity × explicitness**	**31,199**	**.2 (vs. 4)**
6	Familiarity + explicitness	31,204	.008 (vs. 5)
rp—precritical			
1	Literality × explicitness × familiarity	35,390	
2	Literality × familiarity + explicitness	35,385	.8 (vs. 1)
3	Literality × explicitness + familiarity	35,385	.8 (vs. 1)
4	Familiarity × explicitness + literality	35,385	.8 (vs. 1)
5	Literality + familiarity + explicitness	35,383	.8 (vs. 2)
			.8 (vs. 3)
			.5 (vs. 4)
6	Literality + familiarity	35,381	.8 (vs. 5)
7	Literality + explicitness	35,391	.001 (vs. 5)
8	Familiarity + explicitness	35,384	.1 (vs. 5)
**9**	**Familiarity**	**35,382**	**.1 (vs. 6)**
			**.8 (vs. 8)**
10	Intercept	35,390	.001 (vs. 9)
tt—precritical			
1	Literality × explicitness × familiarity	36,874	
2	Literality × familiarity + explicitness	36,871	.3 (vs. 1)
3	Literality × explicitness + familiarity	36,872	.2 (vs. 1)
4	Familiarity × explicitness + literality	36,872	.2 (vs. 1)
5	Literality + familiarity + explicitness	36,870	.3 (vs. 2)
			.8 (vs. 3)
			.8 (vs. 4)
**6**	**Literality + familiarity**	**36,868**	**.5 (vs. 5)**
7	Literality + explicitness	36,876	.004 (vs. 5)
8	Familiarity + explicitness	36,882	<.001 (vs. 5)
9	Literality	36,875	.004 (vs. 6)
10	Familiarity	36,883	<.001 (vs. 6)
fp—critical			
1	Literality × explicitness × familiarity	30,269	
2	Literality × familiarity + explicitness	30,265	.5 (vs. 1)
3	Literality × explicitness + familiarity	30,266	.4 (vs. 1)
4	Familiarity × explicitness + literality	30,264	.6 (vs. 1)
5	Literality + familiarity + explicitness	30,264	.4 (vs. 2)
			.8 (vs. 3)
			.2 (vs. 4)
**6**	**Literality + familiarity**	**30,262**	**.5 (vs. 5)**
7	Literality + explicitness	30391	<.001 (vs. 5)
8	Familiarity + explicitness	30,266	.039 (vs. 5)
9	literality	30,331	<.001 (vs. 6)
10	Familiarity	30,264	.039 (vs. 6)
rp—critical			
1	Literality × explicitness × familiarity	35,502	
2	Literality × familiarity + explicitness	35,498	.4 (vs. 1)
3	Literality × explicitness + familiarity	35,503	.064 (vs. 1)
4	Familiarity × explicitness + literality	35,501	.1 (vs. 1)
5	Literality + familiarity + explicitness	35,501	.03 (vs. 2)
			.8 (vs. 3)
			.2 (vs. 4)
**6**	**Literality × familiarity**	**35,496**	**.9 (vs. 2)**
tt—critical			
1	Literality × explicitness × familiarity	33,709	
2	Literality × familiarity + explicitness	33,703	.9 (vs. 1)
3	Literality × explicitness + familiarity	33,707	.2 (vs. 1)
4	Familiarity × explicitness + literality	33,707	.3 (vs. 1)
5	Literality + familiarity + explicitness	33,705	.047 (vs. 2)
			.9 (vs. 3)
			.5 (vs. 4)
**6**	**Literality × familiarity**	**33,701**	**.9 (vs. 2)**
fp—postcritical			
1	Literality × explicitness × familiarity	39,226	
2	Literality × familiarity + explicitness	39,222	.7 (vs. 1)
3	Literality × explicitness + familiarity	39,221	.8 (vs. 1)
4	Familiarity × explicitness + literality	39,222	.7 (vs. 1)
5	Literality + familiarity + explicitness	39,220	.7 (vs. 2)
			.5 (vs. 3)
			.8 (vs. 4)
6	Literality + familiarity	39,218	.8 (vs. 5)
7	Literality + explicitness	39,219	.3 (vs. 5)
8	Familiarity + explicitness	39,242	<.001 (vs. 5)
**9**	**Literality**	**39,217**	**.3 (vs. 6)**
			**.8 (vs. 7)**
10	Intercept	39,240	<.001 (vs. 9)
rp—postcritical			
1	Literality × explicitness × familiarity	43676	
2	Literality × familiarity + explicitness	43,671	.8 (vs. 1)
3	Literality × explicitness + familiarity	43,671	.7 (vs. 1)
4	Familiarity × explicitness + literality	43,672	.6 (vs. 1)
5	Literality + familiarity + explicitness	43,670	.4 (vs. 2)
			.4 (vs. 3)
			.7 (vs. 4)
**6**	**Literality + familiarity**	**43,668**	**.9 (vs. 5)**
7	Literality + explicitness	43,675	.008 (vs. 5)
8	Familiarity + explicitness	43,682	<.001 (vs. 5)
9	Literality	43,673	.008 (vs. 6)
10	Familiarity	43,680	<.001 (vs. 6)
tt—postcritical			
1	Literality × explicitness × familiarity	41,604	
2	Literality × familiarity + explicitness	41,599	.7 (vs. 1)
3	Literality × explicitness + familiarity	41,599	.6 (vs. 1)
4	Familiarity × explicitness + literality	41,599	.7 (vs. 1)
5	Literality + familiarity + explicitness	41,597	.6 (vs. 2)
			.8 (vs. 3)
			.7 (vs. 4)
**6**	**Literality + familiarity**	**41,598**	**.1 (vs. 5)**
7	Literality + explicitness	41,604	.002 (vs. 5)
8	Familiarity + explicitness	41,609	<.001 (vs. 5)
9	Literality	41,605	.002 (vs. 6)
10	Familiarity	41,610	<.001 (vs. 6)
*Note.* In this table, the fixed-effects structure gets progressively simpler at every step; a *p* value < .05 suggests that the better model fit is the one with the more complex fixed-effects structure out of the two models being compared; similarly, a *p* value > .05 suggests that it is the simpler fixed-effects structure that best describes the data. The fixed-effects structure of the best model fit is in boldface. AIC = Akaike’s information criterion (the smaller the AIC, the better the model fit; Wagenmakers & Farrell, 2004).			

## Footnotes

1 Factors like the frequency or length of the analyses regions were not included as effects in the linear mixed models because these experiments were designed so that we only compared between the same critical words, precritical, and postcritical regions.

## A Selection of Experimental Materials for Experiment 1

**Table:**

Literality	Explicitness	Example scenario
Literal	Explicit	Sam and Tim were in a bar and wanted another round but ran out of money. Knowing how charismatic Tim was, Sam asked him to go and charm the barmaid and get them two free drinks. Tim went up to her, made a few witty jokes and got them the free drinks. “That was masterful!” Sam said to him. They went home.
	Implicit	Sam and Tim were in a bar and wanted another round but ran out of money. They thought it might be a good idea to try their luck at getting two free drinks from the barmaid. Tim went up to her, made a few witty jokes and got them the free drinks. “That was masterful!” Sam said to him. They went home.
Sarcastic	Explicit	Sam and Tim were in a bar and wanted another round but ran out of money. Knowing how charismatic Tim was, Sam asked him to go and charm the barmaid and get them two free drinks. Tim went up to her, made a bad joke, and the barmaid just laughed at him. “That was masterful!” Sam said to him. They went home.
	Implicit	Sam and Tim were in a bar and wanted another round but ran out of money. They thought it might be a good idea to try their luck at getting two free drinks from the barmaid. Tim went up to her, made a bad joke, and the barmaid just laughed at him. “That was masterful!” Sam said to him. They went home.
Literal	Explicit	Paul and Matt went camping together for the weekend. This was Paul’s first time camping, so he asked Matt to bring all the necessary equipment. Matt arrived at the campsite with everything they needed. “You’re equipped so well!” Paul said to him. They were hungry.
	Implicit	Paul and Matt went camping together for the weekend. They hadn’t been outside the city in a long time and were really looking forward to spending some time in nature. Matt arrived at the campsite with everything they needed. “You’re equipped so well!” Paul said to him. They were hungry.
Sarcastic	Explicit	Paul and Matt went camping together for the weekend. This was Paul’s first time camping, so he asked Matt to bring all the necessary equipment. Matt arrived at the campsite with nothing but plastic cutlery. “You’re equipped so well!” Paul said to him. They were hungry.
	Implicit	Paul and Matt went camping together for the weekend. They hadn’t been outside the city in a long time and were really looking forward to spending some time in nature. Matt arrived at the campsite with nothing but plastic cutlery. “You’re equipped so well!” Paul said to him. They were hungry.
Literal	Explicit	Josh and Jane had been living together for over a year now. Josh typically didn’t mind doing housework so one morning she asked him to clean the kitchen. When she came home, Josh had already made the kitchen sparkle clean. “Your help is priceless!” she said to Josh. They then watched TV.
	Implicit	Josh and Jane had been living together for over a year now. Jane was always working late, but today was her turn to clean the kitchen so she was going to do it in the evening. When she came home, Josh had already made the kitchen sparkle clean. “Your help is priceless!” she said to Josh. They then watched TV.
Sarcastic	Explicit	Josh and Jane had been living together for over a year now. Josh typically didn’t mind doing housework so one morning she asked him to clean the kitchen. When she came home, Josh had made an even bigger mess in the kitchen. “Your help is priceless!” she said to Josh. They then watched TV.
	Implicit	Josh and Jane had been living together for over a year now. Jane was always working late, but today was her turn to clean the kitchen so she was going to do it in the evening. When she came home, Josh had made an even bigger mess in the kitchen. “Your help is priceless!” she said to Josh. They then watched TV.
Literal	Explicit	Cara and Eve were in a supermarket doing food shopping and were queuing to pay. Cara asked Eve to carefully put all the food in bags so that they could easily carry them to the car. Eve managed to fit everything in two bags. “You packed them great!” Cara said to her. They drove home.
	Implicit	Cara and Eve were in a supermarket doing food shopping and were queuing to pay. They were preparing a Christmas meal for both their families who were coming to visit. Eve managed to fit everything in two bags. “You packed them great!” Cara said to her. They drove home.
Sarcastic	Explicit	Cara and Eve were in a supermarket doing food shopping and were queuing to pay. Cara asked Eve to carefully put all the food in bags so that they could easily carry them to the car. Eve packed the eggs under the turkey and broke them all. “You packed them great!” Cara said to her. They drove home.
	Implicit	Cara and Eve were in a supermarket doing food shopping and were queuing to pay. They were preparing a Christmas meal for both their families who were coming to visit. Eve packed the eggs under the turkey and broke them all. “You packed them great!” Cara said to her. They drove home.
Literal	Explicit	Lilly and Kim were about to set off on a long journey. Lilly was going to drive and she asked Kim to fill up the petrol tank the night before. When they were about to leave, Lilly saw that Kim remembered to fill up the petrol tank. “Your help is indispensable!” she said to Kim. They drove off.
	Implicit	Lilly and Kim were about to set off on a long journey. They both loved travelling and were now about to go on a hiking trip for their holidays. When they were about to leave, Lilly saw that Kim remembered to fill up the petrol tank. “Your help is indispensable!” she said to Kim. They drove off.
Sarcastic	Explicit	Lilly and Kim were about to set off on a long journey. Lilly was going to drive and she asked Kim to fill up the petrol tank the night before. When they were about to leave, Lilly saw that Kim had forgotten to fill up the petrol tank. “Your help is indispensable!” she said to Kim. They drove off.
	Implicit	Lilly and Kim were about to set off on a long journey. They both loved travelling and were now about to go on a hiking trip for their holidays. When they were about to leave, Lilly saw that Kim had forgotten to fill up the petrol tank. “Your help is indispensable!” she said to Kim. They drove off.

## B Results of Stimulus Norming Tests (Experiment 1)

### Stimulus Norming 1: Familiarity

The purpose of this test was to ensure that all of the target utterances used in this experiment were unfamiliar. A familiarity questionnaire was devised, which contained 178 utterances, presented out of context. Thirteen volunteers were recruited (*M*_age_ = 28 years and 1 month, *SD* = 6 years and 1 month, 7 females and 6 males) and asked to rate how familiar they were with each utterance used sarcastically, on a scale from 1 (*unfamiliar*) to 8 (*familiar*). The 24 unfamiliar utterances for this experiment were then selected from the lowest rated ones and they had a mean of 2.6 (*SD* = 0.5, *min* = 1.38, *max* = 3.62).

### Stimulus Norming 2: Explicitness Manipulation

The purpose of this test was to verify whether there was a clear perceived difference between the explicit and implicit expectation conditions. Fifty-six potential materials were divided into two questionnaires, so that each participant saw only one version of each scenario, either the explicit or the implicit one. Materials were not presented in their entirety, but only up to the second sentence, which either contained or did not contain the expectation. Each scenario was followed by a question, for example for the scenario in Table 1: *“Based only on what you’ve read, does Dean have an expectation for Chloe to suggest an exciting activity for them to do on their final day in Valencia?”* Nineteen volunteers were recruited (*M*_age_ = 26 years and 4 months, *SD* = 5 years and 10 months, 9 females and 10 males) and asked to answer the question by rating each scenario on a scale from 1 (*no such expectation*) to 8 (*clear expectation*). We then selected for use in Experiment 1 the 24 scenarios that had the most extreme difference score between the explicit and the implicit conditions, and conducted a paired-samples *t* test: the explicit expectation condition had significantly higher ratings (*M* = 7.17, *SD* = 0.56) than the implicit expectation condition (*M* = 1.95, *SD* = 0.52), *t*(21) = 29.82, *p* < .001.

### Stimulus Norming 3: Expectation for Sarcasm

The purpose of this test was to verify that, as the implicit display theory predicts, an expectation for sarcasm is increased when an expectation is made explicit in the context and then broken. In other words, for the conditions of the implicit display theory to be met, we would need to observe an interaction between literality and explicitness, such that for sarcastic comments, the expectation for sarcasm is increased in an explicit context compared with an implicit one. We presented 24 participants (*M*_age_ = 25 years and 6 months, *SD* = 10 years and 6 months, 17 females and 7 males) with the experimental scenarios (minus the target comments), and asked them to rate on a scale from 1 (*sarcasm very unlikely*) to 8 (*sarcasm very likely*) how likely they think it is that one character will say something sarcastic to the other character (e.g., for the example in Table 1, *“Do you expect that Dean will now say something sarcastic to Chloe?”*). The materials were randomized and divided into four versions of the questionnaire, so that one participant only saw each scenario in one condition.

The data were analyzed with a linear mixed effects model (*lme4* package in R), and the results suggested that there was a significant interaction between literality and explicitness. Post hoc tests were performed using the *testInteractions* function from the *phia* package in R (where the chi-square is the default post hoc test) and Bonferroni-corrected *p* values are reported. Sarcasm was expected more in explicit scenarios than in implicit ones, only if the expectation in the context was broken (*M*_explicit_ = 5.99, *SEM* = 0.16, *M*_implicit_ = 5.45, *SEM* = 0.17, χ^*2*^(1, *N* = 24) = 5.5, *p* = .038), but equally expected if it was met (*M*_explicit_ = 2.15, *SEM* = 0.14, *M*_implicit_ = 2.37, *SEM* = 0.16, χ^*2*^(1, *N* = 24) = 0.6, *p* = .9). These results support the implicit display theory’s offline prediction, and also reassure us that our materials were optimized for detecting contextual effects.

### Stimulus Norming 4: Naturalness

The purpose of this test was to check whether there were any differences between conditions in how natural the materials sounded. The materials were presented in their entirety to the participants, and they were asked to rate on a scale from 1 (*unnatural*) to 8 (*natural*) how natural sounding or coherent the materials were. The scenarios were divided into four questionnaires, so that each participant only saw each scenario in one condition. Each questionnaire contained six materials from each condition, and additionally six unnatural sounding materials, which were included as controls. These materials had the same structure as the experimental ones, but made no logical sense. Data from 23 participants (*M*_age_ = 18 years and 8 months, *SD* = 8 months, 22 females and 1 male) were analyzed in R (*lme4* package), and results indicated that there was a main effect of literality. Literal scenarios were rated as significantly more natural sounding (*M*_literal_ = 6.32, *SEM* = 0.1) than sarcastic scenarios (*M*_sarcastic_ = 5.40, *SEM* = 0.1). However, when we compared the experimental materials from each condition with the ratings of the unnatural materials (paired-samples *t* tests), all experimental materials were rated as significantly more natural (all *p*s < 0.001) than the unnatural ones (*M*_unnatural_ = 1.75, *SEM* = 0.1).

Although it is reassuring that all the experimental materials were perceived as more natural that the control unnatural scenarios, it is worth noting that the discrepancy in naturalness ratings between literal and sarcastic scenarios might potentially explain some of the reading time differences observed between them. However, it is not possible to control for naturalness because of the very nature of sarcasm—devising a sarcastic interchange that is as natural as a literal equivalent seems unlikely, simply because of the fact that sarcasm is globally more rarely used than literal language.

### Stimulus Norming 5: Sarcasm Rating

The purpose of this test was to verify whether the perception of how sarcastic the target utterance was differed between conditions. The experimental materials were divided into four questionnaires (each scenario was presented in only one condition in each questionnaire). Twenty-four participants (*M*_age_ = 30 years and 11 months, *SD* = 12 years and 2 months, 13 females and 11 males) were asked to rate them on a scale from 1 (*not at all sarcastic*) to 8 (*very sarcastic*) in terms of how sarcastic they thought the final comments were. Data were analyzed in R (*lme4* package), and the results indicated that there was an interaction between literality and explicitness. Post hoc tests showed that literal stories were consistently rated as significantly less sarcastic than sarcastic scenarios, in both explicit (*M*_literal_ = 1.6, *SEM* = 0.08, *M*_sarcastic_ = 6.7, *SEM* = 0.15, χ^*2*^(1, *N* = 24) = 283.7, *p* < .001) and implicit scenarios (*M*_literal_ = 2.1, *SEM* = 0.13, *M*_sarcastic_ = 6.6, *SEM* = 0.14, χ^*2*^(1, *N* = 24) = 235.7, *p* < .001). Literal scenarios were rated as slightly more sarcastic when the expectation in the context was implicit (*M*_implicit_ = 2.1, *SEM* = 0.13) than when it was explicit (*M*_explicit_ = 1.6, *SEM* = 0.08), χ^*2*^(1, *N* = 24) = 7.6, *p* = .01, but there was no difference in how sarcastic scenarios were rated in explicit and implicit contexts (*M*_explicit_ = 6.7, *SEM* = 0.15, *M*_implicit_ = 6.6, *SEM* = 0.14, χ^*2*^(1, *N* = 24) = 0.07, *p* = 1).

Even though literal comments in implicit contexts appeared to be slightly more sarcastic than literal comments in explicit contexts, they did not approach the ratings of sarcastic utterances; hence the literality manipulation was effective regardless of context. Additionally, in this experiment the predictions were not concerned with comparing the reading times between literal utterances in explicit and implicit contexts, but rather between literal and sarcastic utterances in explicit and implicit contexts; therefore, there is no reason to suspect that the slight rating difference between literal explicit and literal implicit scenarios would have any harmful implications for the interpretation of relevant online reading times.

## C Selection of Filler Items for Experiment 1

**Table:**

Two characters + literal negative comment
Barbara and Carlos went into town one afternoon to have a walk. The weather forecast predicted it would be sunny and warm all day long. While they were in town, it suddenly started raining heavily and they didn’t have an umbrella. “The forecast is unreliable!” Barbara said to Carlos. They went home.
Cal was working on a miniature house model he had to build for his architecture course. He was behind schedule and Chris offered to help out by fitting the windows. His work started progressing much faster but he was still pressed for time. “I hate working under stress!” Cal said to Chris. They did their best.
Felicia and Daphne were shopping for a dress. In the last shop they entered, Felicia tried on a red one that she really liked. However the dress was too small for her and there were no other sizes in the shop. “I’m just not lucky today!” Felicia said to Daphne. They went home.
Informative texts
When my car broke down a few nights ago, the first thing I did was to get it out of the road, in a safe place. Once I was there, I called the AA of course. They managed to locate me using the GPS function on my phone. I waited for them for about an hour, but they fixed it quickly when they arrived.
On a Sunday afternoon, we all decided to go grocery shopping in order to make pancakes later in the evening. We had to buy flour, sugar, eggs and a bit of milk. The trouble was, we couldn’t decide what to fill them with, so we got everything: chocolate spread, jam, ice cream and bananas.
Everybody should visit Iceland. It’s so staggeringly beautiful and otherworldly. Everywhere you turn there are glaciers, waterfalls, lava fields, rainbows, streams and mountain ranges. It’s also an ideal destination if you want to see the Northern Lights, especially if you go between February and March.
Two characters + literal positive comment
Harry and Tara were looking to rent a flat in Nottingham. They’d already seen several flats, and had their heart set on one of them. When they called the agency, they found out that their favourite flat was still available. “That’s such great news!” Tara said. They soon moved in.
Greg and Rick had no plans for Friday evening. They rented the “Matrix” trilogy because they had never watched it before. The movies were so good that they stayed awake all night in order to finish them by morning. “These movies are amazing!” Greg said at the end. They went to sleep.
Rose and Nell wanted to buy a present together for a friend’s birthday. They bought her a classical music CD from the new music shop in town. Their friend was very happy about the gift since she had wanted to buy it herself for a while. “This was such a good gift!” Rose said to Nell. They listened to the CD.

## D The *t* Values of Nonsignificant Fixed Effects and *p* Values of Likelihood Ratio Tests (Experiment 1)

[Table-anchor tbl6][Table-anchor tbl7]

## E Selection of Experimental Materials for Experiment 2

**Table:**

Literality	Familiarity	Explicitness	Example scenario
Literal	Familiar	Explicit	Rachel was a model for her friend Nikki’s fashion show. Nikki told Rachel how important this show was and asked her to strive for a perfect look. When she was about to go on stage, Rachel had a flawless make-up and beautifully fixed hair. “Looking good!” Nikki said to Rachel. She was nervous.
		Implicit	Rachel was a model for her friend Nikki’s fashion show. This was an important show for Nikki’s career as many people from the industry were attending. When she was about to go on stage, Rachel had a flawless make-up and beautifully fixed hair. “Looking good!” Nikki said to Rachel. She was nervous.
	Unfamiliar	Explicit	Rachel was a model for her friend Nikki’s fashion show. Nikki told Rachel how important this show was and asked her to strive for a perfect look. When she was about to go on stage, Rachel had a flawless make-up and beautifully fixed hair. “Your look is very chic!” Nikki said to Rachel. She was nervous.
		Implicit	Rachel was a model for her friend Nikki’s fashion show. This was an important show for Nikki’s career as many people from the industry were attending. When she was about to go on stage, Rachel had a flawless make-up and beautifully fixed hair. “Your look is very chic!” Nikki said to Rachel. She was nervous.
Sarcastic	Familiar	Explicit	Rachel was a model for her friend Nikki’s fashion show. Nikki told Rachel how important this show was and asked her to strive for a perfect look. When she was about to go on stage, Rachel’s make-up had worn off and her hair was in a messy state. “Looking good!” Nikki said to Rachel. She was nervous.
		Implicit	Rachel was a model for her friend Nikki’s fashion show. This was an important show for Nikki’s career as many people from the industry were attending. When she was about to go on stage, Rachel’s make-up had worn off and her hair was in a messy state. “Looking good!” Nikki said to Rachel. She was nervous.
	Unfamiliar	Explicit	Rachel was a model for her friend Nikki’s fashion show. Nikki told Rachel how important this show was and asked her to strive for a perfect look. When she was about to go on stage, Rachel’s make-up had worn off and her hair was in a messy state. “Your look is very chic!” Nikki said to Rachel. She was nervous.
		Implicit	Rachel was a model for her friend Nikki’s fashion show. This was an important show for Nikki’s career as many people from the industry were attending. When she was about to go on stage, Rachel’s make-up had worn off and her hair was in a messy state. “Your look is very chic!” Nikki said to Rachel. She was nervous.
Literal	Familiar	Explicit	Daisy had a statistics coursework to do and she was having trouble with it. She asked her friend Iris to have a look since Iris had helped her before with stats. Iris sat with Daisy for an hour and explained to her everything she had to do. “That was really helpful!” Daisy said to her. She needed a high mark.
		Implicit	Daisy had a statistics coursework to do and she was having trouble with it. When she got really confused, she asked her friend Iris to have a look. Iris sat with Daisy for an hour and explained to her everything she had to do. “That was really helpful!” Daisy said to her. She needed a high mark.
	Unfamiliar	Explicit	Daisy had a statistics coursework to do and she was having trouble with it. She asked her friend Iris to have a look since Iris had helped her before with stats. Iris sat with Daisy for an hour and explained to her everything she had to do. “Your help was priceless!” Daisy said to her. She needed a high mark.
		Implicit	Daisy had a statistics coursework to do and she was having trouble with it. When she got really confused, she asked her friend Iris to have a look. Iris sat with Daisy for an hour and explained to her everything she had to do. “Your help was priceless!” Daisy said to her. She needed a high mark.
Sarcastic	Familiar	Explicit	Daisy had a statistics coursework to do and she was having trouble with it. She asked her friend Iris to have a look since Iris had helped her before with stats. Iris wasn’t up for it so she gave a brief explanation that confused Daisy more. “That was really helpful!” Daisy said to her. She needed a high mark.
		Implicit	Daisy had a statistics coursework to do and she was having trouble with it. When she got really confused, she asked her friend Iris to have a look. Iris wasn’t up for it so she gave a brief explanation that only confused Daisy more. “That was really helpful!” Daisy said to her. She needed a high mark.
	Unfamiliar	Explicit	Daisy had a statistics coursework to do and she was having trouble with it. She asked her friend Iris to have a look since Iris had helped her before with stats. Iris wasn’t up for it so she gave a brief explanation that only confused Daisy more. “Your help was priceless!” Daisy said to her. She needed a high mark.
		Implicit	Daisy had a statistics coursework to do and she was having trouble with it. When she got really confused, she asked her friend Iris to have a look. Iris wasn’t up for it so she gave a brief explanation that only confused Daisy more. “Your help was priceless!” Daisy said to her. She needed a high mark.
Literal	Familiar	Explicit	Hugo and Liz had to give a presentation together on Monday as part of the assignment for one of their modules. Hugo needed a high mark so he asked Liz to come well prepared. Liz and Hugo both gave excellent presentations so they got a high mark. “Well that went well!” he said to her. They went home.
		Implicit	Hugo and Liz had to give a presentation together on Monday as part of the assignment for one of their modules. It was the module with the highest number of credits for their degree. Liz and Hugo both gave excellent presentations so they got a high mark. “Well that went well!” he said to her. They went home.
	Unfamiliar	Explicit	Hugo and Liz had to give a presentation together on Monday as part of the assignment for one of their modules. Hugo needed a high mark so he asked Liz to come well prepared. Liz and Hugo both gave excellent presentations so they got a high mark. “Our talk was impeccable!” he said to her. They went home.
		Implicit	Hugo and Liz had to give a presentation together on Monday as part of the assignment for one of their modules. It was the module with the highest number of credits for their degree. Liz and Hugo both gave excellent presentations so they got a high mark. “Our talk was impeccable!” he said to her. They went home.
Sarcastic	Familiar	Explicit	Hugo and Liz had to give a presentation together on Monday as part of the assignment for one of their modules. Hugo needed a high mark so he asked Liz to come well prepared. Hugo did well but Liz was very poorly prepared so they got a low mark. “Well that went well!” he said to her. They went home.
		Implicit	Hugo and Liz had to give a presentation together on Monday as part of the assignment for one of their modules. It was the module with the highest number of credits for their degree. Hugo did well but Liz was very poorly prepared so they got a low mark. “Well that went well!” he said to her. They went home.
	Unfamiliar	Explicit	Hugo and Liz had to give a presentation together on Monday as part of the assignment for one of their modules. Hugo needed a high mark so he asked Liz to come well prepared. Hugo did well but Liz was very poorly prepared so they got a low mark. “Our talk was impeccable!” he said to her. They went home.
		Implicit	Hugo and Liz had to give a presentation together on Monday as part of the assignment for one of their modules. It was the module with the highest number of credits for their degree. Hugo did well but Liz was very poorly prepared so they got a low mark. “Our talk was impeccable!” he said to her. They went home.
Literal	Familiar	Explicit	Amy was almost done writing her final year dissertation when her laptop froze. She knew her friend Rob was very good at computers and could fix it so she asked for his help. He managed to unfreeze the computer and she retrieved her work. “Ahh brilliant!” she said to him. He then went home.
		Implicit	Amy was almost done writing her final year dissertation when her laptop froze. Her friend Rob was in the house at the time and came over to see what had happened. He managed to unfreeze the computer and she retrieved her work. “Ahh brilliant!” she said to him. He then went home.
	Unfamiliar	Explicit	Amy was almost done writing her final year dissertation when her laptop froze. She knew her friend Rob was very good at computers and could fix it so she asked for his help. He managed to unfreeze the computer and she retrieved her work. “You were very helpful!” she said to him. He then went home.
		Implicit	Amy was almost done writing her final year dissertation when her laptop froze. Her friend Rob was in the house at the time and came over to see what had happened. He managed to unfreeze the computer and she retrieved her work. “You were very helpful!” she said to him. He then went home.
Sarcastic	Familiar	Explicit	Amy was almost done writing her final year dissertation when her laptop froze. She knew her friend Rob was very good at computers and could fix it so she asked for his help. He pressed the wrong button, the computer died and all her work was lost. “Ahh brilliant!” she said to him. He then went home.
		Implicit	Amy was almost done writing her final year dissertation when her laptop froze. Her friend Rob was in the house at the time and came over to see what had happened. He pressed the wrong button, the computer died and all her work was lost. “Ahh brilliant!” she said to him. He then went home.
	Unfamiliar	Explicit	Amy was almost done writing her final year dissertation when her laptop froze. She knew her friend Rob was very good at computers and could fix it so she asked for his help. He pressed the wrong button, the computer died and all her work was lost. “You were very helpful!” she said to him. He then went home.
		Implicit	Amy was almost done writing her final year dissertation when her laptop froze. Her friend Rob was in the house at the time and came over to see what had happened. He pressed the wrong button, the computer died and all her work was lost. “You were very helpful!” she said to him. He then went home.
Literal	Familiar	Explicit	Donna was not feeling well and was resting in bed. She had just cleaned her room so she asked her friend Jay to be extra careful not to spill her soup when bringing it over from the kitchen. He brought it to Donna without spilling a single drop. “So careful!” she said to him. He sat down.
		Implicit	Donna was not feeling well and was resting in bed. Her mom had left her a bowl of soup in the kitchen so Donna asked her friend Jay to bring it to her. He brought it to Donna without spilling a single drop. “So careful!” she said to him. He sat down.
	Unfamiliar	Explicit	Donna was not feeling well and was resting in bed. She had just cleaned her room so she asked her friend Jay to be extra careful not to spill her soup when bringing it over from the kitchen. He brought it to Donna without spilling a single drop. “Your balance is so good!” she said to him. He sat down.
		Implicit	Donna was not feeling well and was resting in bed. Her mom had left her a bowl of soup in the kitchen so Donna asked her friend Jay to bring it to her. He brought it to Donna without spilling a single drop. “Your balance is so good!” she said to him. He sat down.
Sarcastic	Familiar	Explicit	Donna was not feeling well and was resting in bed. She had just cleaned her room so she asked her friend Jay to be extra careful not to spill her soup when bringing it over from the kitchen. He managed to spill almost all of it on the floor in her room. “So careful!” she said to him. He sat down.
		Implicit	Donna was not feeling well and was resting in bed. Her mom had left her a bowl of soup in the kitchen so Donna asked her friend Jay to bring it to her. He managed to spill almost all of it on the floor in her room. “So careful!” she said to him. He sat down.
	Unfamiliar	Explicit	Donna was not feeling well and was resting in bed. She had just cleaned her room so she asked her friend Jay to be extra careful not to spill her soup when bringing it over from the kitchen. He managed to spill almost all of it on the floor in her room. “Your balance is so good!” she said to him. He sat down.
		Implicit	Donna was not feeling well and was resting in bed. Her mom had left her a bowl of soup in the kitchen so Donna asked her friend Jay to bring it to her. He managed to spill almost all of it on the floor in her room. “Your balance is so good!” she said to him. He sat down.

## F Results of Stimulus Norming Tests (Experiment 2)

### Stimulus Norming 1: Familiarity

The 48 sarcastic utterances were selected from the 178 possible utterances in the familiarity questionnaire mentioned in Experiment 1. A paired-samples *t* test comparing the ratings of the familiar and unfamiliar utterances showed that the familiar utterances were rated as significantly more familiar (*M*_familiar_ = 6.24, *SD* = 0.86) than the unfamiliar ones (*M*_unfamiliar_ = 2.81, *SD* = 0.83), *t*(47) = 21.12, *p* < .001.

### Stimulus Norming 2: Explicitness Manipulation

The data from the test from Experiment 1 were also used to select the materials for the second experiment. The 48 contexts were chosen from the 56 possible contexts in the expectation questionnaire, by selecting the ones that had the largest difference in ratings between the explicit and the implicit conditions. A paired-samples *t* test was then conducted in order to compare between the ratings for the explicit and the implicit expectation conditions. The explicit expectations condition had significantly higher ratings (*M*_explicit_ = 7.02, *SD* = 0.74) than the implicit expectations condition (*M*_implicit_ = 3.08, *SD* = 1.41), *t*(47) = 16.7, *p* < .001.

### Stimulus Norming 3: Expectation for Sarcasm

The next three tests had exactly the same structure and procedure as the ones in Experiment 1. Twenty-four participants completed the test (*M*_age_ = 27 years and 1 month, *SD* = 8 years, 15 females and 9 males). The data were analyzed in R (*lme4* package), and the results suggested that there was a significant interaction between literality and explicitness on the expectation for sarcasm ratings. Sarcasm was expected more in explicit scenarios than in implicit ones, only if the expectation from the context was broken (*M*_explicit_ = 5.59, *SEM* = 0.12, *M*_implicit_ = 5.12, *SEM* = 0.12, χ^*2*^(1, *N* = 24) = 10.2, *p* = .002), but equally expected if it was met (*M*_explicit_ = 2.17, *SEM* = 0.09, *M*_implicit_ = 2.46, *SEM* = 0.11, χ^*2*^(1, *N* = 24) = 3.6, *p* = .1). These results suggest, as in Experiment 1, that the experimental materials are suitable to test the predictions of the implicit display theory.

### Stimulus Norming 4: Naturalness

Twenty-four participants took part (*M*_age_ = 18 years and 6 months, *SD* = 6 months, 20 females and 4 males). The data were analyzed in R (*lme4* package), and the results indicated that there were two main effects, of literality and familiarity. Literal scenarios were rated as more natural (*M*_literal_ = 6.62, *SEM* = 0.06) than sarcastic ones (*M*_sarcastic_ = 5.59, *SEM* = 0.07), and also familiar scenarios were rated as more natural (*M*_familiar_ = 6.27, *SEM* = 0.07) than unfamiliar ones (*M*_unfamiliar_ = 5.94, *SEM* = 0.07). However, as in Experiment 1, scenarios in each condition were rated as significantly more natural than the unnatural controls (all *p*s < 0.001, *M*_unnatural_ = 1.55, *SEM* = 0.06).

### Stimulus Norming 5: Sarcasm Rating

Forty-eight participants took part (M_age_ = 25 years and 3 months, *SD* = 7 years and 9 months, 25 females and 23 males). The data were analyzed in R (*lme4* package), and the results indicated that there was an interaction between literality and familiarity. Post hoc tests showed that sarcasm ratings were significantly higher for sarcastic scenarios than for literal ones, irrespective of familiarity (*M*_sarcastic-familiar_ = 7.07, *SEM* = 0.06, *M*_literal-familiar_ = 1.59, *SEM* = 0.07, χ^*2*^(1, *N* = 48) = 665, *p* < .001, *M*_sarcastic-unfamiliar_ = 6.88, *SEM* = 0.07, *M*_literal-unfamiliar_ = 1.95, *SEM* = 0.07, χ^*2*^(1, *N* = 48) = 561.8, *p* < .001). Familiar scenarios were rated marginally higher than unfamiliar ones in sarcastic scenarios (*M*_sarcastic-familiar_ = 7.07, *SEM* = 0.06, *M*_sarcastic-unfamiliar_ = 6.88, *SEM* = 0.07, χ^*2*^(1, *N* = 48) = 5.5, *p* = .038), and rated the same in literal scenarios (*M*_literal-familiar_ = 1.59, *SEM* = 0.07, *M*_literal-unfamiliar_ = 1.95, *SEM* = 0.07, χ^*2*^(1, *N* = 48) = 1.4, *p* = .5). The literality effect was completely expected: sarcastic comments were rated as significantly more sarcastic than literal scenarios. The difference between familiar and unfamiliar sarcastic comments, statistically significant yet extremely small in magnitude (a difference of 0.19 rating points), was perhaps also not surprising, seeing how by definition, readers are more exposed to and more accustomed to hearing the familiar comments as sarcastic rather than the unfamiliar ones.

## G The *t* Values of Nonsignificant Fixed Effects and *p* Values of Likelihood Ratio Tests (Experiment 2)

[Table-anchor tbl8][Table-anchor tbl9]

## References

[c1] Baayen R. H., Davidson D. J., & Bates D. M. (2008). Mixed-effects modelling with crossed random effects for subjects and items. Journal of Memory and Language, 59, 390–412. 10.1016/j.jml.2007.12.005

[c2] Barr D. J., Levy R., Scheepers C., & Tily H. J. (2013). Random effects structure for confirmatory hypothesis testing: Keep it maximal. Journal of Memory and Language, 68, 255–278. 10.1016/j.jml.2012.11.001 PMC3881361 24403724

[c3] Bryant G. A. (2012). Is verbal irony special? Language and Linguistic Compass, 6, 673–685. 10.1002/lnc3.364

[c4] Burgers C., van Mulken M., & Schellens P. J. (2011). Finding irony: An introduction of the Verbal Irony Procedure (VIP). Metaphor and Symbol, 26, 186–205. 10.1080/10926488.2011.583194

[c5] Calmus A., & Caillies S. (2014). Verbal irony processing: How do contrast and humour correlate? International Journal of Psychology, 49, 46–50. 10.1002/ijop.12003 24811722

[c6] Campbell J. D., & Katz A. N. (2012). Are there necessary conditions for inducing a sense of sarcastic irony? Discourse Processes, 49, 459–480. 10.1080/0163853X.2012.687863

[c7] De Grauwe S., Swain A., Holcomb P. J., Ditman T., & Kuperberg G. R. (2010). Electrophysiological insights into the processing of nominal metaphors. Neuropsychologia, 48, 1965–1984. 10.1016/j.neuropsychologia.2010.03.017 20307557 PMC2907657

[c8] Filik R., Leuthold H., Wallington K., & Page J. (2014). Testing theories of irony processing using eye-tracking and ERPs. Journal of Experimental Psychology: Learning, Memory, and Cognition, 40, 811–828. 10.1037/a0035658 24548324

[c9] Filik R., & Moxey L. M. (2010). The on-line processing of written irony. Cognition, 116, 421–436. 10.1016/j.cognition.2010.06.005 20598677

[c10] Frisson S., & Pickering M. J. (1999). The processing of metonymy: Evidence from eye movements. Journal of Experimental Psychology: Learning, Memory, and Cognition, 25, 1366–1383. 10.1037/0278-7393.25.6.1366 10605827

[c11] Gibbs R. W. (2000). Irony in talk among friends. Metaphor and Symbol, 15, 5–27. 10.1080/10926488.2000.9678862

[c12] Gibbs R. W. Jr. (1986). On the psycholinguistics of sarcasm. Journal of Experimental Psychology: General, 115, 3–15. 10.1037/0096-3445.115.1.3

[c13] Gibbs R. W. Jr. (1999). Interpreting what speakers say and implicate. Brain and Language, 68, 466–485. 10.1006/brln.1999.2123 10441189

[c14] Gibbs R. W. Jr., & Colston H. L. (2012). Interpreting figurative meaning. New York, NY: Cambridge University Press 10.1017/CBO9781139168779

[c15] Giora R. (1995). On irony and negation. Discourse Processes, 19, 239–264. 10.1080/01638539509544916

[c16] Giora R. (1997). Understanding figurative and literal language: The graded salience hypothesis. Cognitive Linguistics, 8, 183–206. 10.1515/cogl.1997.8.3.183

[c17] Giora R. (2003). On our mind: Salience, context and figurative language. New York, NY: Oxford University Press 10.1093/acprof:oso/9780195136166.001.0001

[c18] Giora R., & Fein O. (1999). Irony: Context and salience. Metaphor and Symbol, 14, 241–257. 10.1207/S15327868MS1404_1

[c19] Giora R., Fein O., Kaufman R., Eisenberg D., & Erez S. (2009). Does an “ironic situation” favour an ironic interpretation? In Brône G. & Vandaele J. (Eds.), Cognitive poetics: Goals, gains, and gaps (pp. 383–399). New York, NY: Mouton de Gruyter.

[c20] Giora R., Fein O., Laadan D., Wolfson J., Zeituny M., Kidron R., . . . Shaham R. (2007). Expecting irony: Context versus salience-based effects. Metaphor and Symbol, 22, 119–146. 10.1080/10926480701235346

[c21] Giora R., Fein O., & Schwartz T. (1998). Irony: Graded salience and indirect negation. Metaphor and Symbol, 13, 83–101. 10.1207/s15327868ms1302_1

[c22] Giora R., Livnat E., Fein O., Barnea A., Zeiman R., & Berger I. (2013). Negation generates nonliteral interpretations by default. Metaphor and Symbol, 28, 89–115. 10.1080/10926488.2013.768510

[c23] Grice H. P. (1975). Logic and conversation In Morgan J. & Cole P. (Eds.), Syntax and semantics: Vol. 3 Speech acts (pp. 41–58). New York, NY: Academic Press.

[c24] Hancock J. T. (2004). Verbal irony use in face-to-face and computer-mediated conversations. Journal of Language and Social Psychology, 23, 447–463. 10.1177/0261927X04269587

[c25] Ivanko S. L., & Pexman P. M. (2003). Context incongruity and irony processing. Discourse Processes, 35, 241–279. 10.1207/S15326950DP3503_2

[c26] Kaakinen J. K., Olkoniemi H., Kinnari T., & Hyönä J. (2014). Processing of written irony: An eye movement study. Discourse Processes, 51, 287–311. 10.1080/0163853X.2013.870024

[c27] Kowatch K., Whalen J. M., & Pexman P. M. (2013). Irony comprehension in action: A new test of processing for verbal irony. Discourse Processes, 50, 301–315. 10.1080/0163853X.2013.799934

[c28] Kreuz R. J., & Glucksberg S. (1989). How to be sarcastic: The echoic reminder theory of verbal irony. Journal of Experimental Psychology: General, 118, 374–386. 10.1037/0096-3445.118.4.374

[c29] Matuschek H., Kliegl R., Vasishth S., Baayen H., & Bates D. (2015). Balancing Type I error and power in linear mixed models. Retrieved from http://arxiv.org/abs/1511.01864.

[c30] Peleg O., Giora R., & Fein O. (2001). Salience and context effects: Two are better than one. Metaphor and Symbol, 16, 173–192. 10.1080/10926488.2001.9678894

[c31] Pexman P. M. (2008). It’s fascinating research: The cognition of verbal irony. Current Directions in Psychological Science, 17, 286–290. 10.1111/j.1467-8721.2008.00591.x

[c32] Pexman P. M., Ferretti T. R., & Katz A. N. (2000). Discourse factors that influence online reading of metaphor and irony. Discourse Processes, 29, 201–222. 10.1207/S15326950dp2903_2

[c33] Pexman P. M., Whalen J. M., & Green J. J. (2010). Understanding verbal irony: Clues from interpretation of direct and indirect ironic remarks. Discourse Processes, 47, 237–261. 10.1080/01638530902959901

[c34] R Core Team (2013). R: A language and environment for statistical computing. Vienna, Austria: R Foundation for Statistical Computing Retrieved from http://www.R-project.org/

[c35] Spotorno N., & Noveck I. A. (2014). When is irony effortful? Journal of Experimental Psychology: General, 143, 1649–1665. 10.1037/a0036630 24773194

[c36] Utsumi A. (2000). Verbal irony as implicit display of ironic environment: Distinguishing ironic utterances from nonirony. Journal of Pragmatics, 32, 1777–1806. 10.1016/S0378-2166(99)00116-2

[c37] Utsumi A. (2005). Stylistic and contextual effects in irony processing In Forbus K., Gentner D., & Regier T. (Eds.), Proceedings of the 26th Annual Conference of the Cognitive Science Society (pp. 1369–1375). Austin, TX: Cognitive Science Society.

[c38] Wagenmakers E.-J., & Farrell S. (2004). AIC model selection using Akaike weights—Notes and comments. Psychonomic Bulletin & Review, 11, 192–196. 10.3758/BF03206482 15117008

